# Developmental deficits and staging of dynamics of age associated Alzheimer’s disease neurodegeneration and neuronal loss in subjects with Down syndrome

**DOI:** 10.1186/s40478-021-01300-9

**Published:** 2022-01-04

**Authors:** Jerzy Wegiel, Michael Flory, Izabela Kuchna, Krzysztof Nowicki, Jarek Wegiel, Shuang Yong Ma, Nanbert Zhong, Teresa Wierzba Bobrowicz, Mony de Leon, Florence Lai, Wayne P. Silverman, Thomas Wisniewski

**Affiliations:** 1 Department of Developmental Neurobiology, NYS Institute for Basic Research in Developmental Disabilities, 1050 Forest Hill Road, Staten Island, NY 10314 USA; 2 New York State Institute for Basic Research in Developmental Disabilities (IBR), Staten Island, NY USA; 3 Institute of Psychiatry and Neurology, Warsaw, Poland; 4 Department of Radiology, Weill Cornell Medicine, New York, NY USA; 5 Department of Neurology, Massachusetts General Hospital, Harvard Medical School, Boston, MA USA; 6 Department of Pediatrics, Irvine Medical Center, University of California, Irvine, CA USA; 7 Center for Cognitive Neurology, Departments of Neurology, Pathology and Psychiatry, NYU Grossman School of Medicine, New York, NY 10016 USA

**Keywords:** Down syndrome, Alzheimer’s disease, Clinicopathological staging, Developmental neuronal deficits, Neuronal loss, Neurofibrillary degeneration, β-amyloidosis, Lewy bodies, TDP-43 neurodegeneration, Stereology

## Abstract

The increased life expectancy of individuals with Down syndrome (DS) is associated with increased prevalence of trisomy 21–linked early-onset Alzheimer’s disease (EOAD) and dementia. The aims of this study of 14 brain regions including the entorhinal cortex, hippocampus, basal ganglia, and cerebellum in 33 adults with DS 26–72 years of age were to identify the magnitude of brain region–specific developmental neuronal deficits contributing to intellectual deficits, to apply this baseline to identification of the topography and magnitude of neurodegeneration and neuronal and volume losses caused by EOAD, and to establish age-based staging of the pattern of genetically driven neuropathology in DS. Both DS subject age and stage of dementia, themselves very strongly correlated, were strong predictors of an AD-associated decrease of the number of neurons, considered a major contributor to dementia. The DS cohort was subclassified by age as pre-AD stage, with 26–41-year-old subjects with a full spectrum of developmental deficit but with very limited incipient AD pathology, and 43–49, 51–59, and 61–72-year-old groups with predominant prevalence of mild, moderately severe, and severe dementia respectively. This multiregional study revealed a 28.1% developmental neuronal deficit in DS subjects 26–41 years of age and 11.9% AD-associated neuronal loss in DS subjects 43–49 years of age; a 28.0% maximum neuronal loss at 51–59 years of age; and a 11.0% minimum neuronal loss at 61–72 years of age. A total developmental neuronal deficit of 40.8 million neurons and AD-associated neuronal loss of 41.6 million neurons reflect a comparable magnitude of developmental neuronal deficit contributing to intellectual deficits, and AD-associated neuronal loss contributing to dementia. This highly predictable pattern of pathology indicates that successful treatment of DS subjects in the fourth decade of life may prevent AD pathology and functional decline.

**Supplementary Information:**

The online version contains supplementary material available at 10.1186/s40478-021-01300-9.

## Introduction

Down syndrome (DS), caused by trisomy of chromosome 21 [31, 42], is the most common form of chromosomal abnormality; its prevalence increased between 1979 and 2003 from 9.0 to 11.8 per 10,000 live births. The life expectancy of the DS population increased from 9 years in 1929 to 61.1 years for men with DS and 57.8 years for women with DS in 2000, due to advances in medical care, and advances in societal support [79, 107]. Increase in life expectancy resulted in an increase of the DS population [26, 66]. However, positive changes with a significant increase of life expectancy in the DS population are associated with an increased risk of early-onset Alzheimer’s disease (AD) and dementia, exceeding 50% prior to 60 years of age and continuing to rise thereafter [52].

### Developmental abnormalities in the brain of DS subjects and developmental intellectual deficits

That DS, the leading genetic cause of intellectual disability, is characterized by reduced brain size and a reduced number of neurons throughout development, points to altered neurogenesis as a major determinant of structural and functional anomalies. In the 17th to 21st gestational week (GW), the germinal zone in the ventricle wall of DS fetuses reveals a reduced proliferation rate of progenitors of neurons involved in brain cortical mantle formation [11]. Deficits in neuron number and brain volume deficits were detected both in affected fetuses and children [11, 62, 63]. Neuropathological studies indicate that in DS, developmental defects of neurogenesis might be the main factor contributing to multiregional brain hypoplasia and functional abnormalities including cognitive impairment [57].

### AD neuropathology and dementia in DS

Individuals with an additional copy of chromosome 21 (trisomy 21) and DS are affected by both brain developmental abnormalities leading to intellectual deficits and early onset of Alzheimer-type neuropathology, including β-amyloidosis with diffuse and fibrillar plaque formation and amyloid angiopathy, and neurofibrillary degeneration with abnormal tau hyperphosphorylation and neurofibrillary tangle (NFT) formation common in individuals with DS older than 40 years of age [9, 30, 49, 70, 104, 105]. In DS subjects, further progression of neurodegeneration and neuronal loss is associated with the early onset of functional decline and dementia.

### Aims

Estimation of the impact of DS developmental defects on neurogenesis leading to neuronal developmental deficit is critical for distinguishing the topography and severity of developmental deficits from mechanisms of neuronal loss caused by AD and other age-associated mechanisms of neuronal death.

Therefore, the first aim of this morphometric study of the brain of DS subjects was to estimate brain structure/neuronal population–specific developmental neuronal deficits in 14 brain structures and their cytoarchitectural subdivisions in 4 subjects in the pre-AD stage of life (26–41 years of age).

Developmental deficits were determined by estimation of the difference between the volume of selected structures and the total number of neurons in these structures in DS subjects 26–41 years of age and age matched control subjects. The second aim was to test the hypothesis that both age and stage of dementia are predictors of structural and functional decline in DS/AD population.

The third aim was to establish trajectories of neurofibrillary degeneration, neuronal loss and volume reduction, and β-amyloidosis in an AD-positive DS cohort subdivided into those 43–49 years of age with a prevalence of mild AD, a 51–59 years of age group with a prevalence of moderate/moderately severe AD, and a 61–72 years of age group with a prevalence of severe AD (Table 1).

**Table 1:** Clinical and neuropathological characteristics of DS subjects

#	Case #	Sex	Age (y)	Cause of death	Intellectual (IQ) deficit	Epilepsy	APOE allele	Dementia	Brain weight (g)	BraakStage: NFTs	Thal Phase: Aβ plaques
1	207–93	M	26					No signs	1,160	I	0
2	292–00	M	28	Gastrointest. hemorrhage				No signs	1,320	I	5
3	37–98	M	41		Severe, 28			Mild	1,130	II	5
4	M16-03	F	41	Peritonitis	Severe, 20			No signs	926	IV	5
5	256–00	F	43	Pneumonia	Profound, 17		3/4	No signs	990	V	5
6	257–00	M	48	Pneumonia	Profound, 12			No signs	940	IV	5
7	M9-04	M	48	Pneumonia	Severe, 27	+	4/4	Mild	920	VI	5
8	24–98	M	49		Mild, 50			Mild	1,100	III	5
9	1–98	M	51		Moderate, 41			Mod/ severe	840	VI	5
10	511–02	M	51	Pneumonia				Mod/ severe	954	VI	5
11	512–02	M	52	Cardiac infarct				Mod/ severe	1,194	VI	5
12	482–97	F	54	Pneumonia	Profound, 19	+	3/3		900	VI	5
13	181–00	M	54	Pneumonia	Moderate, 42			Mild	1,060	VI	5
14	55–03	M	54	Sepsis			3/3		774	VI	5
15	M18-03	F	56	SUDEP	Mild, 50	+		Mild	656	VI	5
16	M1-05	F	56	Asp. pneumonia	Moderate, 43	+		Severe	822	VI	5
17	M2-09	M	56	Asp. pneumonia	Mild, 58	+	3/3	Mod/ severe	762	VI	5
18	M2-06	M	56	Cardiac infarct			4/4		1,114	VI	5
19	M10-04	F	57	Pneumonia	Moderate, 42	+	3/3	Mod/ severe	960	VI	5
20	M3-05	F	57	Cardiac failure			3/4		696	VI	5
21	M1-06	M	57				3/3		1,000	VI	5
22	552–97	F	57		Profound, 15		3/3	Mod/ severe	790	VI	5
23	615–97	F	59	Cardiac failure	Profound, 18		4/3	Severe	740	VI	5
24	649–97	M	59		Profound, 5			Severe	1,060	VI	5
25	M11-04	M	59	Pneumonia	Moderate, 43		3/4	Severe	1,028	VI	5
26	M2-05	M	61	Pneumonia	Severe, 27			Severe	1,208	VI	5
27	M3-06	F	61	Subdural cerebellar hemorrhage			2/3	Severe	1,102	VI	5
28	37–99	M	63	Pneumonia	Severe, 20	+		Severe	700	VI	5
29	650–97	F	65		Severe, 26		4/4	Severe	830	VI	5
30	258–00	M	65	Pneumonia			3/3	Mod/ severe	900	VI	5
31	256–99	M	67					Severe	700	VI	5
32	M18-07	M	71	Multiple white matter infarcts	Severe, 26	+	3/3	Mod/ severe	960	VI	5
33	483–97	F	72						1,100	VI	5

M18-07 diagnosed with mosaic trisomy. SUDEP = sudden unexpected death in epilepsy

Longitudinal clinical-pathological studies indicate that AD dementia is a cumulative result of multiple pathologies that contribute to lowering the threshold for clinically evident AD dementia that include non-AD cerebrovascular disease (CVD), AD-related common amyloid angiopathy [28], Lewy bodies, TDP-43 proteinopathy, and hippocampal sclerosis [35]. Therefore, the fourth aim was to expand estimates of neuronal loss as a correlate of functional decline, including AD dementia, with estimates of the contribution of various proteinopathies (neurofibrillary degeneration, α-synucleinopathy with Lewy bodies, aggregation of abnormally phosphorylated TDP-43 protein, and amyloid angiopathy).

Our overall goal is to establish an age-based subclassification of AD pathology in early-onset AD in trisomic subjects with DS corresponding to the clinicopathological staging of the dynamics of pathology in late-onset AD (LOAD), distinguishing mild cognitive impairment (MCI)/mild AD (Functional Assessment Staging (FAST 3–4), moderate/moderately severe AD (FAST 5–6), and severe AD (FAST 7) [69, 94].

The study defined developmental volume and neuronal count deficits in 14 structures in a pre-AD group of DS subjects 26–41 years of age which delineate the difference between the baseline for onset and progression of genetically driven EOAD in DS and the baseline for sporadic LOAD. Regression analysis revealed genetically driven EOAD in the DS cohort, with strong correlations among age, stage of dementia, structure volume, neuronal count, and percentage of neurons with NFTs in almost all examined brain regions, confirming the hypothesis that both age and stage of dementia are strong predictors of structural and functional decline in the DS/AD population.

## Materials and methods

### Materials

This study of developmental abnormalities and staging of neurodegeneration and neuronal loss in the brain of subjects with trisomy 21 and DS is the third part of our comparative study of human brain aging and loss of the neuronal reserve [93], and clinicopathological staging of LOAD [94]. Methods of tissue preservation, processing, cutting, staining, and immunostaining as well as stereological estimation of brain structure volume, number of neurons and neurons with NFTs, and amyloid load (%) were standardized to facilitate comparison of changes in neurologically normal control subjects, subjects with LOAD, and trisomic subjects diagnosed with DS and EOAD. In this report, the material and methods described in detail in two previous reports [93, 94] are presented in an abbreviated format.

To establish the pattern of developmental neuronal deficits and AD staging of neurodegeneration and neuronal loss in DS, 33 brain hemispheres of DS subjects 26–72 years of age, 64% of whom were males and 36%, females, were examined (Table 1). Samples of frozen cortex from the frontal lobe of 16 DS subjects were used for DNA extraction and APOE genotyping (LGC Genomics LLC, Alexandria, MN, USA). One or more ApoE ɛ4 alleles, considered a significant risk factor for dementia and β-amyloidosis [12, 65, 73, 76], were detected in 37.5% of the 16 cases genotyped (4/4 3x, 3/4 3x), whereas the promotion of longevity and the protection of patients with DS from dementia by APOE ɛ2 [73] was found in only one case. Diagnosis of Down syndrome trisomy 21, including one case of mosaic trisomy was extracted from subjects’ medical records. Intellectual deficits ranged from mild (14%), to moderate (24%), severe (33%) and profound (29%). A history of seizures reported in eight DS subjects (24%) in this postmortem-examined cohort was comparable to that (20.6%) detected in individuals with DS 36 years of age and older in a study of comorbidities of 602 individuals diagnosed with DS [80].

In the 26–41-year-old group, 75% of individuals with DS had no signs of dementia, whereas in the 43–49-year-old group 50% of subjects were free of dementia and 50% were diagnosed with mild AD dementia. In the 51–59-year-old group, 50.6% of individuals had moderately severe dementia, over 16% had mild dementia, and 33% had severe dementia. In the oldest group, 61–72 years of age, the percentage of subjects diagnosed with moderately severe dementia decreased to 28.6%, and severe dementia increased to 71.4%. In this postmortem study, the diagnosis of dementia was based on case records and caregivers’ reports. In the DS cohort, the most common causes of death were pneumonia and cardiac failure. In other cases, brain infarct, subdural cerebellar hemorrhage, gastrointestinal hemorrhage, peritonitis, sepsis, and sudden unexpected epilepsy related death (SUDEP) were reported.

The control group consisted of 20 subjects, 25–78 years of age, with no records of functional decline or dementia who were characterized in our previous report [94]. This was postmortem study without contact with subject and caregiver. This postmortem study of control group was based on donations to the New York State Brain and Tissue Bank for Developmental Disabilities and Aging at the New York State IBR, Tissue Bank for Developmental Disorders of the National Institute of Child Health and Human Development at the University of Maryland, and the Alzheimer’s Disease Research Center at the New York University Grossman School of Medicine.The information about absence of functional decline was recovered from anonymized medical records. Cases with not convincing records of absence of functional decline were not included in this study.

### Methods of neuropathological and stereological studies

For neuropathological studies, brain hemispheres were fixed for several months with 10% buffered formalin. Sequential 10-mm-thick coronal slabs cut with custom made macrotome were washed, dehydrated in ascending concentrations of ethyl alcohol, infiltrated with polyethylene glycol 400 (PEG; Sigma, St. Louis, MO, USA) and with PEG 1000 for two weeks, and embedded in fresh PEG 1000. Tissue blocks were cut with heavy-duty sliding microtome LEICA SM 2500 (Leica Microsystem Nussloch GmbH, Germany) into serial 50-µm-thick sections, and enumerated free-floating sections were stored in 70% ETOH [93].

For morphological and morphometric studies, serial equidistant free-floating sections were stained with cresyl violet (CV) and used for delineation of borders of examined structures, estimation of brain structure, and anatomical subdivision volume and number of neurons. The percentage of examined structure volume occupied with monoclonal antibody (mAb) 4G8–immunopositive amyloid-β deposits (amyloid load) was determined in sections pretreated with formic acid and incubated with mouse mAb 4G8 [36]. Sections for evaluation of neurofibrillary degeneration were pretreated with alkaline phosphatase (Sigma, type VII-L) and incubated with Tau-1 antibody [23, 93].

### Staging of topographic expansion of neurofibrillary degeneration and β-amyloidosis in DS/AD cohort

Staging of topographic expansion of neurofibrillary degeneration in the examined DS/AD cohort was based on examination of coronal hemispheric sections immunostained with mouse monoclonal Tau-1 antibody. Raters applied original Braak criteria [7], distinguishing stage I with neurofibrillary degeneration in the transentorhinal cortex and entorhinal cortex (EC), stage II with spread of degeneration to the hippocampus, stage III with pathology in the temporal cortex, stage IV with expansion of tau pathology to other regions of neocortex, stage V with degeneration in the visual association cortex, and stage VI with degeneration in the primary visual cortex.

For staging of β-amyloidosis, sections immunostained with mAb4G8 were examined, and amyloidosis was classified by using the Thal et al. criteria [84] distinguishing five phases of topographic expansion of Aβ-deposition, including plaque formation limited to the neocortex (phase 1) and expansion of plaque formation to the hippocampus and amygdala (phase 2); to the diencephalic nuclei, including the putamen, the caudate nucleus (CN), the substantia innominata, and magnocellular basal complex (MBC) of cholinergic nuclei (phase 3); to brainstem nuclei (phase 4); and to the cerebellum (phase 5).

### TDP43 aggregation, Lewy bodies, and amyloid angiopathy

The focus of this subproject was the amygdala, which is the first brain structure to be affected by TDP43 aggregation and has a percentage of affected neurons that increases with age [8, 32]. The free-floating 50-µm-thick sections were immunostained with rabbit polyclonal phosphorylation independent anti-TDP-43 antibody 10,782–1-AP diluted 1:500 (Protein Tech, Rosemont, Chicago, IL, USA). Estimation of TDP-43 degeneration was paralleled with estimation of the prevalence and severity of Lewy body neurodegeneration as a marker of α-synucleinopathy. The percentage of neurons with Lewy bodies was estimated in sections immunostained with mouse mAb 4B12 detecting α-synuclein (1:500; MA1-90,346, Thermo Fisher Scientific, IL, USA). To calculate the percentage of neurons with inclusions, the number of neurons estimated in adjacent cresyl violet–stained sections was considered as 100%. The number of vascular profiles (n/mm^2^) immunopositive for mAb 4G8 was determined in the EC, CA1, and subiculum as well as in the molecular layer of the cerebellar cortex with prominent amyloidosis in the wall of arteries and arterioles and massive diffuse amyloid deposits.

### Stereological and statistical analysis

To establish a multiregional pattern of developmental and age- and AD-associated changes, 14 brain regions were examined, including structures found to be involved in intellectual deficits, memory loss, and a broad spectrum of functional declines. The study of the memory system included early and severely affected EC (all layers), and most affected islands of stellate neurons in the second layer delineated by using Amaral and Insausti anatomical criteria [2]. To compare patterns of pathology in different cytoarchitectural subdivisions of the hippocampus, cornu Ammonis (CA) sectors CA1, 2, 3, and 4 and the subiculum were examined by using the anatomical criteria of Rosene and van Hoesen [71] and Duvernoy [19]. The study included the dopaminergic system with all parts of the substantia nigra (SN) [20], and the acetylcholinergic system with all subdivisions of the MBC [56, 88]. Volume and number of neurons were estimated in the entire amygdala and thalamus. Examination of the cerebellum included the volume of the molecular and granule layers to determine the total number of Purkinje cells known as free of neurofibrillary degeneration and to estimate amyloid load in the molecular layer, as well as pathology in the dentate nucleus affected by neurofibrillary degeneration but without amyloid plaques.

### Sampling scheme

The procedures and parameters applied to estimates of the volume and number of neurons of each of 14 structures/anatomical subdivision are summarized in Additional file 1: Table 1. An optical fractionator systemic random sampling scheme from Stereo Investigator (MicroBrightField Inc, Vermont, USA) was applied. The mean number of equidistant sections examined per structure/per case ranged from 6 sections (amygdala four nuclei, entire thalamus, substantia nigra pars compacta and reticulata), 8 sections (all layers of the entorhinal cortex, islands of stellate neurons in the entorhinal cortex; measurements of the cerebellar molecular and granule cell layer volume used for estimation of the total number of Purkinje cells), 11 sections (for estimation of the volume and number of neurons in the magnocellular basal complex including Ch1-Ch4, and the dentate nucleus); 12 sections for stereological estimates of the subiculum, to 14 sections to estimate the volume of the caudate nucleus and the number of small neurons in the caudate nucleus. The efficiency of systematic sampling has been proposed by Gundersen et al. [25]. The number of counted neurons in individual structures ranged from 74 in the islands in the entorhinal cortex to 862 Purkinje cells. The grid size and size of the virtual counting space were adjusted to individual structure size and shape to reduce the variation reflected in the coefficient of error (CE) and standard deviation. The Scheaffer coefficient of error, decreasing with an increasing number of counted neurons, number of counting frames, and number of sections, was maintained close to the required 0.05 (MicroBrightField, Inc.) and ranged from 0.05 to 0.09 except for very small islands of stellate neurons in the entorhinal cortex with CE = 0.19.

The volume of regions of interest and their cytoarchitectural subdivisions, the number of neurons with and without NFTs, and amyloid load were estimated with unbiased stereological methods [95] (Additional file 1: Table 1 ). The number of unaffected neurons and neurons with NFTs was estimated by using a 40 × objective and final magnification of 1450x. The numerical density of Purkinje cells was estimated per volume of molecular and granular layer of the cerebellar cortex by using a 20 × objective and 720 × final magnification. Neuronal estimates were supported with stereology software (Stereo Investigator, Micro Brightfield Bioscience, Inc., Willistone, VT, USA).

Comparisons of structure volumes, number of neurons, NFTs, and amyloid load between groups were analyzed in t-tests adjusted for unequal variances when required. Linear regressions on age, stage of dementia, their interaction were used to estimate the relative degrees of association of age and stage of dementia with neuropathological characteristics. Pearson correlations are unadjusted, and significance levels are not adjusted for multiple comparisons. Analyses were performed in version 16 of Stata [22].

## Results

### Braak/Braak staging of neurofibrillary degeneration detected with mAb Tau-1 and Thal et al. staging of topographic expansion of β-amyloidosis detected with mAb 4G8

**Fig. 1:**
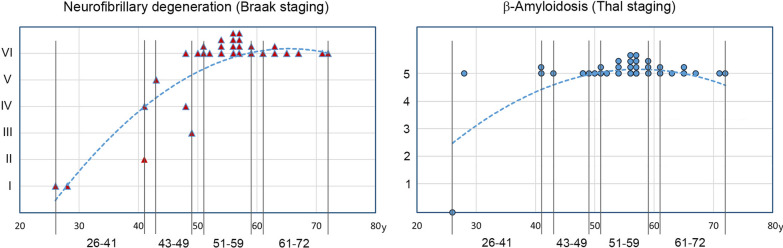
Braak staging of neurofibrillary degeneration and Thal staging of β-amyloidosis. Examination of serial equidistant coronal sections stained with mAb Tau-1 detecting NFTs revealed that topographic expansion of neurofibrillary degeneration in subjects with DS results in progression from Braak stage I in 25- and 26-year-old subjects to stage VI in all subjects 51 years of age and older. The pattern of amyloid plaque distribution corresponds to Thal phase 5 in all subjects 28 years of age and older. Observed pattern reflects completion of topographic expansion at the beginning of fourth decade

**Fig. 2:**
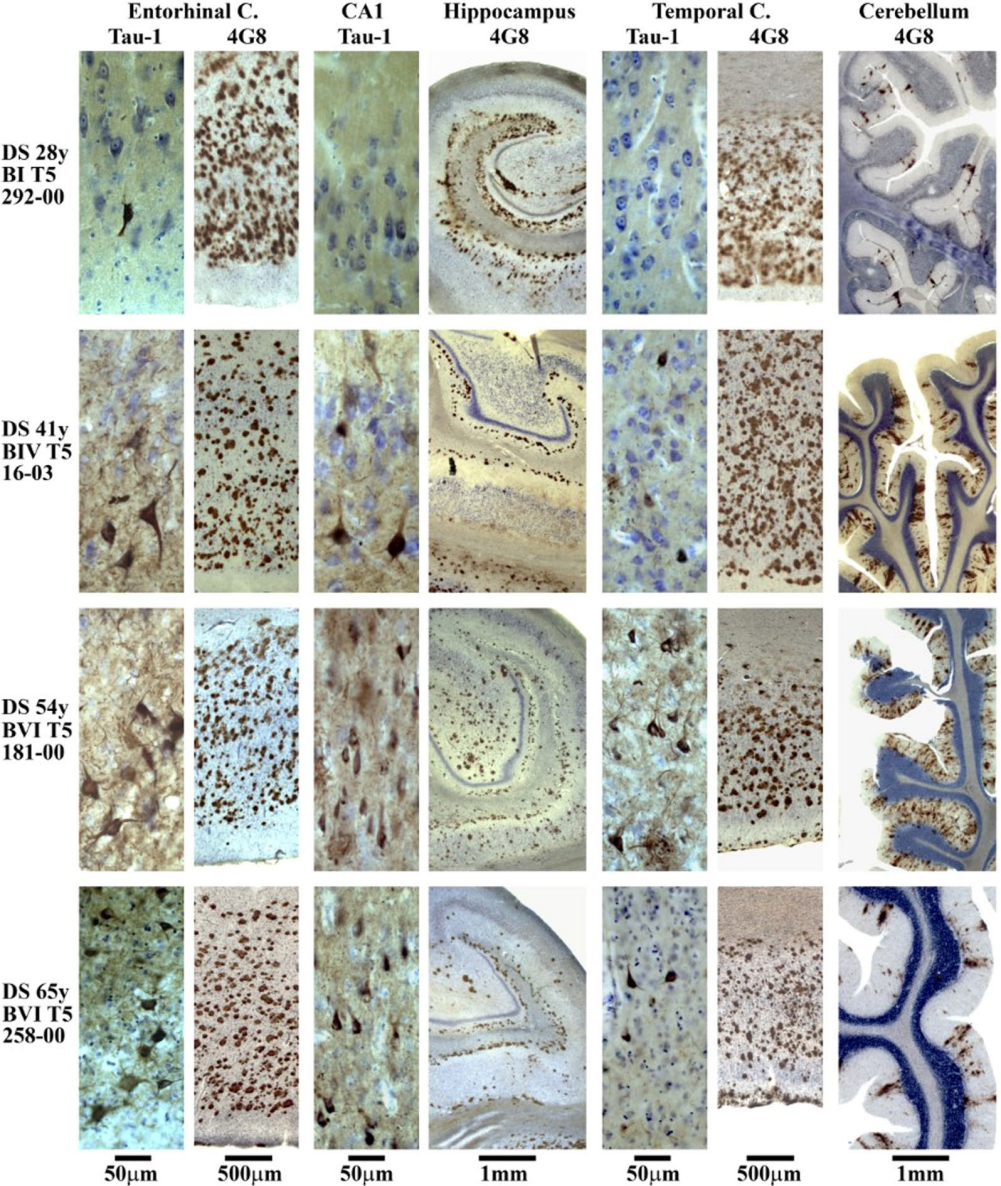
Immunostaining with mAb 4G8 detecting β amyloid in plaques and mAb Tau-1 detecting abnormally phosphorylated tau protein in NFTs illustrates staging of β amyloidosis and neurofibrillary degeneration in four individuals with Down syndrome including 28 years old subject with no signs of dementia, Braak stage I with a few NFTs in the entorhinal cortex but significant β amyloidosis in the entorhinal cortex, hippocampus, temporal, and cerebellar cortex corresponding to final Thal phase 5. Progression of neurofibrillary degeneration observed in 41-year-old DS subject with no signs of dementia (Braak stage IV and Thal phase 5), and in 54-year-old DS subject with mild dementia (Braak stage VI and Thal phase 5), reflects topographic expansion and more severe neurofibrillary degeneration and β amyloidosis in the entorhinal cortex, hippocampus, and temporal cortex. Severe β amyloidosis affects molecular layer of the cerebellar cortex in 41- and 54-year-old subjects. In 65-year-old DS subject diagnosed with moderate severe dementia the number of neurons, including neurons with NFTs, and amyloid load in the temporal and cerebellar cortex decrease but pathology still meets criteria for diagnosis of Braak stage VI and Thal phase 5

In DS subjects 26–41 years of age classified in this study as in the pre-AD stage and used for the estimation of developmental deficit without or with very limited AD neuronal loss, Braak stage I of early neurofibrillary degeneration with a few affected neurons was found in two individuals 26 and 28 years of age, whereas in two subjects 41 years of age, the pathology corresponded to stages II and IV (Table 1, Figs. 1 and 2). In DS subjects 43–49 years of age classified as having mild AD, Braak stages III, IV, V, and VI of neurofibrillary degeneration were detected. In all DS subjects 51 or more years of age with prevalence of mild, moderately severe, and severe AD, the topographic pattern and severity of neurofibrillary degeneration corresponded to Braak final stage VI. β-amyloidosis was absent in a subject 26 years of age, whereas in all DS subjects 28 years of age or older, distribution of amyloid deposits matched that in Thal final phase 5.

### Strong correlations between age as well as stage of dementia and declining number of neurons in DS/AD population

**Fig. 3:**
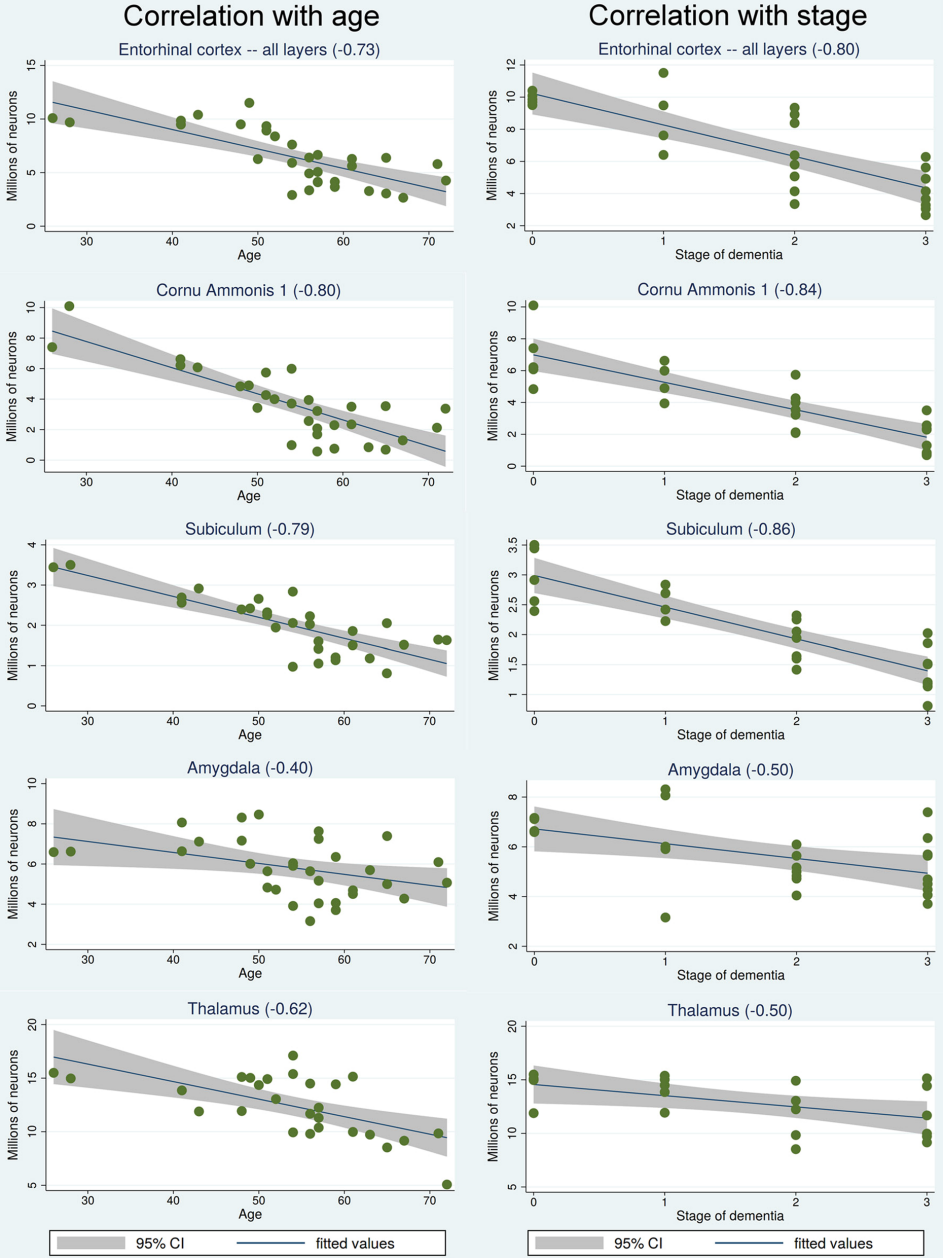
Correlations between age and stage of dementia and declining number of neurons. The panel of regression analysis-based graphs reveals that both stage of dementia and age are equally strong predictors of the decreasing number of neurons in the entorhinal cortex, CA1, subiculum, amygdala, and thalamus in the DS/AD cohort. Staging of dementia: 0—no signs; 1—mild, 2- moderately severe; 3—severe

Table 2 shows that the volume of the majority of examined structures, including the entorhinal cortex, the cornu Ammonis and subiculum, as well as the amygdala, thalamus, substantia nigra and magnocellular basal complex of DS subjects correlates with age as well as with stage of dementia. A regression analysis-based panel of graphs (Fig. 3) reveals that the pattern of correlations between age and a decreasing number of neurons in the entorhinal cortex, CA1, subiculum, amygdala and thalamus is very similar to the pattern of correlations between stage of dementia (no dementia, mild, moderate, and severe dementia) and the decline of the number of neurons in examined structures in the DS/AD population.

**Table 2:** Correlations with age and dementia stage of structure volume, neuron number, NFTs number, NFT percentage and amyloid load (%)

Structure	Correlation with age of					Correlation with dementia stage of				
	Structure volume	Neuron number	NFT number	NFT %	Amyloid load (%)	Structure volume	Neuron number	NFT number	NFT %	Amyloid load (%)
Entorhinal cortex – all layers	− 0.67***	−0.73***	0.40*	0.69***	0.10	−0.79***	− 0.80***	0.41*	0.76***	− 0.01
Entorhinal cortex − islands	−0.84***	− 0.78***	0.07	0.81***	−	−0.85***	− 0.84***	− 0.07	0.80***	
CA1	−0.72***	− 0.80***	0.29	0.71***	0.33	−0.76***	− 0.84***	0.20	0.76***	0.40*
CA2	− 0.42*	− 0.59***	0.57**	0.56**	0.28	−0.60**	− 0.73***	0.45*	0.58**	0.51*
CA3	0.23	− 0.48**	0.57***	0.51**	0.26	−0.27	− 0.51***	0.69***	0.66***	0.38
CA4	− 0.22	− 0.55**	0.34	0.57**	0.34	−0.14	− 0.54**	0.40	0.71***	0.42*
Subiculum	0.69***	0.79***	0.46*	0.62***	0.28	−0.83***	− 0.86***	0.38	0.59**	0.27
Amygdala	− 0.49**	− 0.40*	0.46**	0.58***	0.04	−0.44*	− 0.55**	0.42*	0.52**	− 0.10
Thalamus	−0.58**	0.62***	0.53**	0.49*	0.50*	−0.49*	− 0.50*	0.33	0.42	0.44*
Substantia nigra	−0.72***	0.72***	0.25	0.42*	0.46*	−0.62**	− 0.66***	0.26	0.41	0.44*
Magnocellular basal complex	−0.53**	−0.63***	0.39*	0.46*	0.51**	−0.62**	− 0.70***	0.48*	0.61**	0.34
CN	−0.45**	−0.31	0.30	0.28	0.66***	−0.55**	− 0.52**	0.31	0.29	0.50*
DN	0.11	−0.04	− 0.46*	0.40	–	−0.08	− 0.03	0.72***	0.71***	
Purkinje cells	–	−0.38	–	–	–		−0.31			

^*^p < 0.05; **p < 0.01; ***p < 0.001

### Interactions between age and stage of dementia

**Fig. 4:**
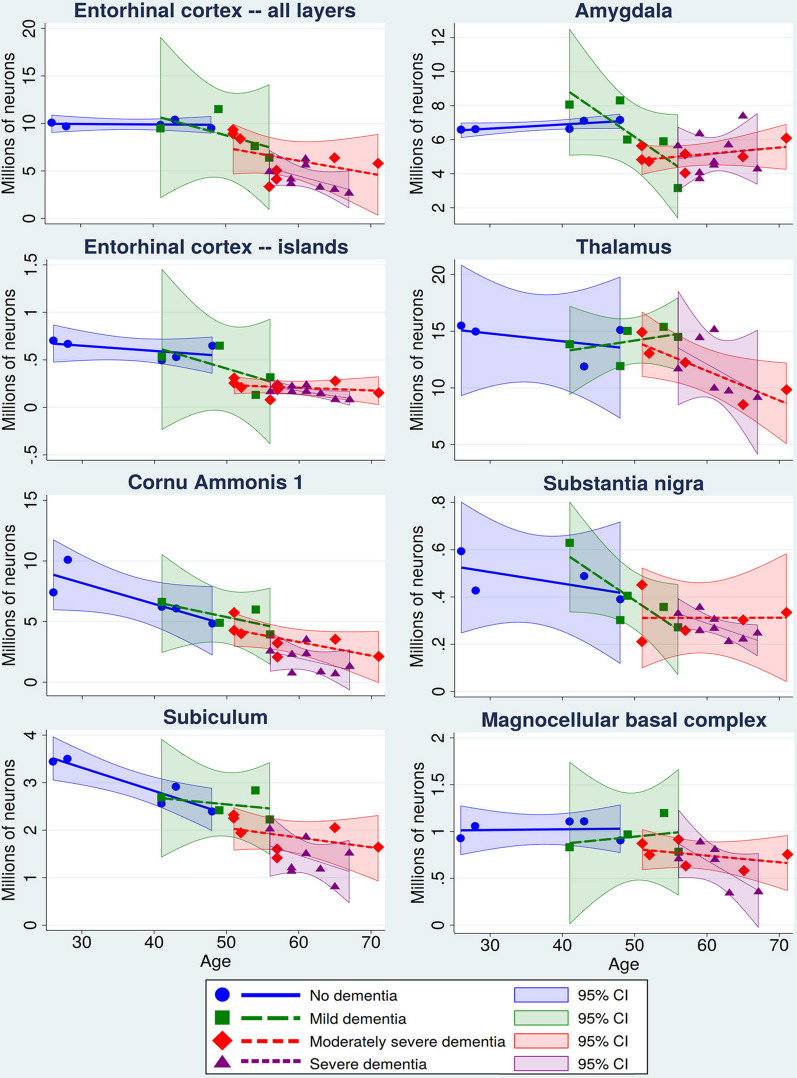
Interactions of age and stage of dementia. Interactions are illustrated by a combination of scatterplots of age-associated decline in neuronal number and segmentation of the period from 26 to 72 years of age with staging of dementia in the DS cohort, including subjects with no signs of dementia and with mild, moderately severe, and severe dementia. Each of the four stages is defined by a regression-fitted line and a 95% confidence interval. Interactions are demonstrated in the entorhinal cortex (all layers), entorhinal cortex islands of stellate neurons, CA1, subiculum, amygdala, thalamus, substantia nigra and magnocellular basal complex

Figure 4 demonstrates interactions between age and stage of dementia using graphic segmentation of age-associated neuronal decline by dementia staging in the DS cohort. The eight selected structures illustrate similarities of age and stage of dementia-associated decrease in the number of neurons in the memory system (all EC layers, EC Islands, CA1, and subiculum), amygdala and thalamus with a major contribution to functional deterioration in AD, and two structures in the neurotransmitter systems involved in functional deterioration, including the dopaminergic system (substantia nigra) and cholinergic system (magnocellular basal complex). Graphs demonstrate segmentation of the continued process of age-associated neuronal loss for almost forty years by clinical staging identifying structure-specific patterns of neuronal decline in no dementia, mild, moderately severe, and severe dementia stages. Graphs show that despite genetic control of AD onset and progression, individual differences result in a broad range of age of diagnosis of dementia stages. No signs of dementia stage were diagnosed in 26–48-year-old subjects (mean age 37.2 y); mild dementia in individuals 41–56 years of age (mean 49.6 y); moderately severe dementia in 51–71-year-old subjects (mean 57.5 y); and severe dementia in 56–67-year-old DS subjects (mean 61.1 y) (Table 1).

### Topography and magnitude of developmental brain structures’ volume and neuronal deficits in pre-AD DS subjects 26–41 years of age

**Fig. 5:**
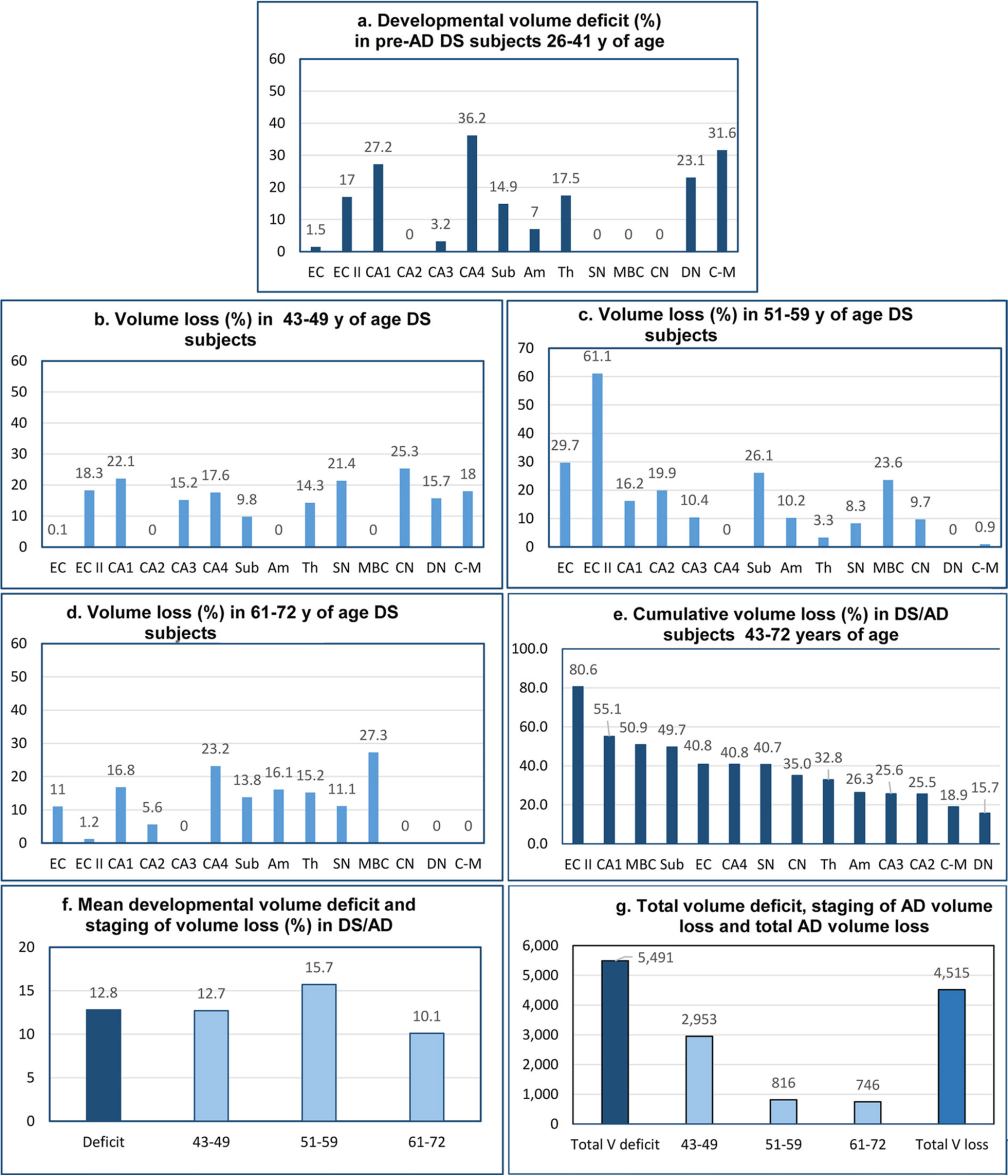
Developmental volume deficits and staging of AD-associated volume loss. Developmental volume deficits in 26- to 41-year-old DS subjects show striking regional differences, with no detectable deficits in four of 14 structures, but with deficits ranging from 1.5% in the entorhinal cortex to 36.2% in CA4 (**a**). AD-associated volume losses are also region-specific and are more uniform in the fourth decade than in the fifth and sixth decades (**b-d**). Cumulative volume loss reveals a gradient of susceptibility to regional atrophy, with ECII, CA1, and MBC the most affected (**e**). The mean developmental volume deficit in 14 examined structures (12.8%) is similar to mean volume losses in each of three stages of AD (**f**). The total developmental volume deficit (5,491 mm^3^) exceeds insignificantly the total volume loss (4,515 mm^3^) produced by three decades of AD pathology (**g**)

Examination of 14 brain structures revealed striking regional differences ranging from no deficit in CA2, SN, MBC, and CN to 17.0% volume deficits in the EC II, 27.2% in CA1, 36.2% in CA4, 14.9% in the subiculum, 7.0% in the amygdala, and 17.5% in the thalamus. High developmental volume deficits were also detected in the cerebellum, including a 23.1% deficit in the dentate nucleus and a 31.6% volume deficit in the molecular layer of the cerebellar cortex (Table 3) (Fig. 5). The detected pattern of developmental deficits in the EC, hippocampal formation, amygdala, thalamus, and cerebellum indicates that in contrast to subjects with sporadic AD (sAD), in subjects with DS, structures developing AD-associated neurodegeneration and neuronal losses are already structurally modified by a mean 12.8% developmental volume deficit and 5,491 mm^3^ total volume deficit in the 14 examined brain regions.

**Table 3:** Estimated developmental volume deficit in DS cohort

Structure/subdivision	Volume (mm^3^) in controls 25–43 y of age (N = 6) Mean and (SD)	Volume (mm^3^) in DS subjects 26–41 y (N = 4) Mean and (SD)	Volume deficit (mm^3^) difference between controls 25-43y of age and DS subjects 26–41 y of age	Volume deficit (%)	Volume deficit *p* <
Entorhinal cortex -all layers	622.0 (85.6)	612.7 (120.6)	9.3	1.5	ns
Entorhinal cortex—islands	38.9 (6.4)	32.2 (8.1)	6.7	17.0	ns
CA1	456.2 (49.6)	332.2 (95.4)	123.9	27.2	ns
CA2	30.8 (4.4)	42.8 (15.9)	–	–	–
CA3	44.7 (10.9)	43.2 (20.9)	1.4	3.2	ns
CA4	102.7 (19.0)	65.5 (21.8)	37.2	36.2	0.042
Subiculum	184.2 (19.6)	156.8 (34.4)	27.5	14.9	ns
Amygdala	452.0 (65.1)	420.5 (57.1)	31.5	7.0	ns
Thalamus	3,639.7 (169.6)	3002.7 (339.2)	637.1	17.5	ns
Substantia nigra	110.0 (26.8)	126.3 ( 12.2)	–	–	–
MBC	123.3 (9.1)	154.6 (19.3)	–	–	–
Caudate nucleus	1,939.2 (197.4)	2271.5 (285.5)	–	–	–
Cerebellum molecular layer	14,348.0 (2,412)	9813.0 (303.3)	4,535.0	31.6	ns
Dentate nucleus	351.6 (62.4)	270.0 (99.0)	81.6	23.1	ns
			Cumulative developmental volume deficit: 5,491.2 mm^3^	Mean developmental volume deficit: 12.8%	

MBC = magnocellular basal complex

**Fig. 6:**
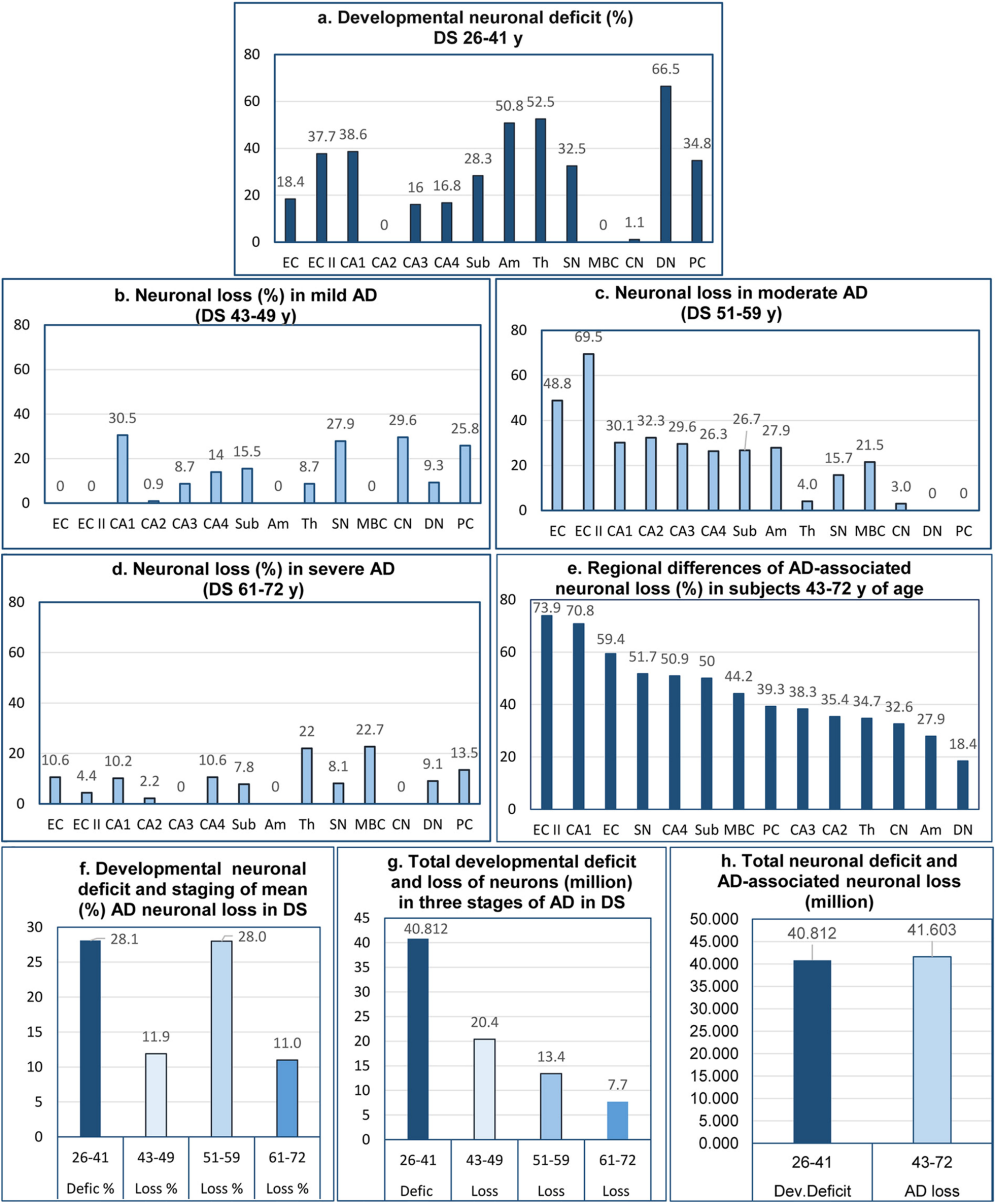
Developmental neuronal deficits and staging of AD-associated neuronal loss. Graphs characterize region-specific developmental neuronal deficits (**a**) as well as region-specific neuronal loss in the fourth, fifth, and sixth decades of life of DS subjects (**b, c, d**, respectively). Cumulative neuronal loss reveals the gradient of different susceptibilities to neurodegeneration and neuronal death reflected in neuronal losses, ranging from 18.4% in the dentate nucleus to 73.9% in the second layer of the entorhinal cortex (**e**). Developmental deficit of neurons in examined structures is estimated as 28.1%, whereas percentage of lost neurons increases from 11.9% in fourth decade to top level (28.0%) in fifth decade and decreases to bottom level (11.0%) in sixth decade of life of DS subjects (**f**). The measure of dynamic of AD-associated neuronal loss is the top level of neuronal loss (20.4 million) in the early stage (fourth decade) and the decline of neuronal loss to 13.4 million in the fifth decade and further decline to the lowest level (7.7 million) in the sixth decade (**g**). Surprisingly, the estimated total developmental deficit of neurons (40.8 million) contributing to developmental intellectual deficits is almost identical with the total neuronal loss (41.6 million) contributing to AD-associated dementia in DS subjects (**h**)

To expand understanding of developmental deficits and AD-associated volume losses, the numbers of neurons and neuronal deficits were estimated. Developmental neuronal deficit in 14 structures in the brain of DS subjects, estimated as the difference in the number of neurons between neurologically normal 25- to 33-year-old control subjects and 26- to 41-year-old DS subjects in the pre-AD stage, is the baseline for delineation of difference between developmental deficits and staging of AD-associated neurodegeneration and neuronal loss in DS subjects 43–72 years of age (Table 4) (Fig. 6). Deficits in CA1 (38.6%), no deficit in CA2, but 16.0% deficit in CA3, 16.8% in CA4, and 28.3% in the subiculum reflect differing severities of developmental abnormalities within parts of the same anatomical/functional complex. A 34.8% deficit of Purkinje cells and 66.5% deficit of neurons in the dentate nucleus reflect the severity of developmental deficits in the cerebellum. Deficits in the EC and the second layer of the EC (18.4% and 37.7%, respectively) as well as in CA1 (38.6%), CA3 16.0%), and CA4 (16.8%) reflect pre-AD developmental deficits in structures most susceptible to early and severe AD neurodegeneration and neuronal loss. Regional diversity within the dopaminergic and cholinergic systems is reflected in a 32.5% neuronal deficit in the SN but the absence of a detectable deficit in the MBC. The topography and severity of developmental deficits reflect both a broad spectrum of contributions of neuronal network developmental pathology to functional deficits as well as pre-AD modifications affecting the course of AD pathology and the onset and course of functional decline including dementia in DS subjects.

**Table 4:** Estimated developmental neuronal deficit—difference between controls 25–33 years of age and DS subjects 26–41 years of age (pre-AD stage)

Structure/subdivision	Number of neurons in controls 25–33 y of age (N = 4) Mean and (SD)	Number of neurons in DS subjects 26–41 y of age (N = 4)Mean and (SD)	Neuronal deficit: difference between controls 25–33 y and DS subjects 26–41 y of age	Neuronal deficit %	Neuronal deficit *p* <
Entorhinal cortex—all layers	11,990,000 (1,774,815)	9,779,100 (255,051)	2,210,900	18.4	ns
Entorhinal cortex—islands	964,696 (149,494)	600,288 (100,952)	364,408	37.7	ns
CA1	12,347,870 (723,418)	7,584,266 (1,745,386)	4,763,604	38.6	ns
CA2	1,344,755 (102,483)	1,375,511 (435,724)	0	0	
CA3	1,265,037 (411,514)	1,062,288 (467,225)	202,749	16.0	ns
CA4	1,329,732 (105,406)	1,105,866 (159,035)	223,866	16.8	ns
Subiculum	4,256,000 (397,748)	3,049,650 (492,839)	1,206,350	28.3	ns
Amygdala	14,192,266 (2,976,788)	6,978,730 (724,797)	7,213,356	50.8	ns
Thalamus	31,086,400 (1,755,888)	14,774,176 (837,618)	16,312,224	52.5	ns
Substantia nigra	815,726 (179,515)	550,053 (107,611)	265,673	32.5	ns
MBC	865,795 (87,266)	981,577 (124,910)	0	0	
Caudate nucleus	41,884,561 (4,548,630)	41,434,112 (4,788,237)	450,449	1.1	ns
Purkinje cells	21,845,212 (499,288)	14,247,128 (2,946,322)	7,598,084	34.8	ns
Dentate nucleus	1,528,995 (267,727)	510,829 (26,959)	1,018,166	66.5	ns
			Cumulative developmental neuronal deficit: 40,812,681	Mean developmental neuronal deficit: 28.1%	

MBC = magnocellular basal complex

### Topography and magnitude of AD-associated volume loss in DS subjects

A 12.7% mean volume loss was detected in 43- to 49-year-old DS subjects (Table 5, Fig. 5), but this loss was extended by an additional 15.7% mean volume loss in 51–59-year-old DS subjects with a prevalence of moderately severe AD and by an additional 10.1% loss in 61–72-year-old subjects with a prevalence of severe AD**.** Examination of 14 brain structures in three age groups revealed strikingly different volume losses in individual structures and different stages of AD, including 18.3% volume loss in the ECII in the 43–49-year-old group, 61.1% loss in the 51–59-year-old group, but only 1.2% in the 61–72-year-old group with severe AD. The opposite trend is observed in the amygdala, with undetectable volume loss in the fourth decade of life, but 10.2% loss in the 5th decade and 16.1% loss in the sixth decade. Structures with undetectable volume developmental deficit reveal prominent losses in the 4^th^ decade, including the SN (21.4%), CN (25.3%), and cerebellar molecular layer (18%), with a decrease in the percentage of volume loss in the 5^th^ and 6^th^ decades.

**Table 5:** Estimated AD-associated volume loss (mm^3^ and %) in three age/AD stages in DS

Structure/ subdivision	Volume loss in 43- to 49-y- old DS subjects, N = 4 (no signs or mild dementia)			Volume loss in 51-to 59-y-old DS subjects, N = 17 (prevalence of moderately severe dementia)			Volume lost in 61-to 72- y- old DS subjects, N = 8 (prevalence of severe dementia)		
	Volume- difference between 26–41 y and 43–49 y old	Volume loss (%)	*p* <	Volume difference between 43–49 y and 51–59 y old	Volume loss (%)	*p* <	Volume difference between 51–59 y and 61–72 y old	Volume loss (%)	*p* <
Entorhinal cortex.—all layers	0.3	0.1	ns	181.8	29.7	0.0001***	67.6	11.0	0.036*
Entorhinal cortex—islands	5.9	18.3	ns	19.7	61.1	0.0001***	0.4	1.2	ns
CA1	73.6	22.1	0.009**	53.9	16.2	0.036*	56.0	16.8	0.005**
CA2	0.0	0.0	–	8.5	19.9	0.014*	2.4	5.6	ns
CA3	6.6	15.2	ns	4.5	10.4	ns	–	0.0	ns
CA4	11.5	17.6	0.045*	–	–	–	15.2	23.2	ns
Subiculum	15.4	9.8	0.022*	41.0	26.1	0.002**	21.7	13.8	0.036*
Amygdala	–	–	–	43.0	10.2	0.050*	67.6	16.1	0.005**
Thalamus	427.9	14.3	ns	101.1	3.3	ns	459.1	15.2	0.036*
Substantia nigra	27.1	21.4	0.0009***	10.6	8.3	0.035*	14.0	11.1	0.034*
MBC	–	–	–	36.5	23.6	0.009**	42.3	27.3	0.007 **
Caudate nucleus	575.3	25.3	ns	222.0	9.7	ns	–	0.0	ns
Cerebellar cortex: molecular and granule cells layer	1767.0	18.0	0.033*	93.3	0.9	ns	–	0.0	ns
Dentate nucleus	42.5	15.7		–	–	–	–	–	–
	Cumulative volume loss in 43–49 y of age DS subjects: 2,953.1 mm^3^	Mean volume loss: 12.7%		Cumulative volume loss in 51–59 y of age DS subjects: 816.0 mm^3^	Mean volume loss: 15.7%		Cumulative volume loss in 61–72 y of age DS subjects: 746.3 mm^3^	Mean volume loss: 10.1%	

Structure-specific AD-associated volume loss (%) calculated in all three stages/age groups in reference to baseline volume in DS subjects 26–41 years of age (100%) who are affected by developmental deficits but not, or in limited range, by pre-AD pathology. *p < 0.05; **p < 0.01; ***p < 0.001

MBC = magnocellular basal complex

Volume of the molecular and granule cells layer of the cerebellar cortex was measured to estimate total number of Purkinje cells dispersed in the border zone between these layers

A measure of structure-specific differences of AD pathology is the cumulative volume loss in AD in DS subjects 43–72 years of age, varying from 40.8% in all layers of the EC to 80.6% in the EC’s second layer of stellate neurons; from 25.6% in CA3 to 55.1% in CA1, 26.3% in the amygdala, 32.8% in the thalamus, 35.0% in the CN, 40.7% in the SN, and 50.9% in the MBC (Fig. 5).

The cumulative mean percentages of volume loss for all examined structures in 43- to 49-, 51- to 59-, and 61- to 72-year-old DS subjects are relatively similar (12.7%, 15.7%, and 10.1%, respectively). However, estimates of AD-associated total volume loss (mm^3^) reveal differences, with the most severe volume loss in the youngest group (43–49 years of age) (2,953 mm^3^), a 3.6 × lower volume loss in 51- to 59-year-old subjects (816 mm^3^), and the lowest volume loss (746 mm^3^) in 61- to 72-year-old subjects with a prevalence of severe AD (Fig. 5).

The difference in volume between developmental deficits and AD-associated changes is reflected in a ratio between the 5,491 mm^3^ total developmental volume deficit in 26- to 41-year-old DS subjects and the 4,515 mm^3^ volume loss during the 29-year duration of AD in DS subjects 43–72 years of age. This pattern reflects the contribution of developmental pathological processes to lifetime intellectual deficits and cognitive decline and dementia in 43- to 72-year-old subjects diagnosed with AD.

### Staging of AD-associated neuronal loss in DS subjects

Neuronal loss in mild AD in DS was estimated as the difference between the number of neurons in pre-AD DS subjects 26–41 years of age and DS/AD subjects 43–49 years of age. To estimate neuronal loss in each age stage of AD, the difference in the number of neurons between 43–49 and 51–59 years of age, and 51–59 and 61–72 years of age was calculated. The percentage of neuronal loss was calculated in reference to the number of neurons in the pre-AD DS subjects 26–41 years of age (100%) (Table 6).

**Table 6:** Estimated AD-associated neuronal loss in 43–49-yr-old DS subjects (loss per day and per million neurons/day)

Structures/ subdivisions	Number of neurons in 26- to 41-y-old DS subjects N = 4, Mean and (SD)	Number of neurons in 43- to 49-y-old DS subjects N = 4, Mean and (SD)	Neurons lost (vs. 26-to 41-y- old DS subjects)	Neurons lost (%)	*P* <	Number of neurons lost/day	Number of neurons lost/million/day
Entorhinal cortex—all layers	9,779,100 (255,051)	10,470,333 (1,003,992)	0.0	0.0		0	
Entorhinal cortex—islands	600,288 (100,952)	609,481 (68,719)	0.0	0.0		0	
CA1	7,584,266 (1,745,386)	5,271,466 (699,001)	2,312,800	30.5	ns	487.4	64.3
CA2	1,375,511 (435,724)	1,363,407 (350,731)	12,104	0.9	ns	2.6	1.9
CA3	1,062,288 (467,225)	969,837 (225,781)	92,451	8.7	ns	19.5	18.3
CA4	1,105,866 (159,035)	950,304 (255,206)	155,562	14.0	ns	32.8	29.6
Subiculum	3,049,650 (492,839)	2,575,933 (293,733)	473,717	15.5	ns	99.8	32.7
Amygdala	6,978,730 (724,797)	7,150,380 (940,312)	0.0	0.0		0	0
Thalamus	14,774,176 (837,618)	13,488,304 (1,821,914)	1,285,871	8.7	ns	271.0	18.3
Substantia nigra	550,053 (107,611)	396,519 (76,289)	135,534	27.9	ns	32.4	58.8
MBC	981,577 (124,910)	994,389 (104,089)	0.0	0.0		0	0
Caudate nucleus	41,434,112 (4,788,237)	29,151,928 (7,214,428)	12,282,183	29.6	0.03*	2,588.4	62.5
Purkinje cells	14,247,128 (2,946,322)	10,569,930 (720,445)	3,677,198	25.8	ns	775.0	54.4
Dentate n	510,829 (26,959)	463,150 (138,022)	47,679	9.3	ns	10.0	19.7
			Total loss: 20,475,099 neurons	Mean loss: 11.9%		Mean loss 308.4/d Total loss 4,318.9/d	Mean loss 25.7/million/day

Losses per day and per million neurons per day were calculated using the 13-year (4,745-day) difference in mean age between the groups as the dominator

^*^p < .05

### Incipient stage of neuronal loss in 43- to 49-year-old DS subjects

The prominent features of neuronal loss in 43- to 49-year-old DS subjects with prevalence of mild AD were the striking differences between examined structures (Table 6, Fig. 6). Neuronal loss in this age group had features of the incipient stage with a surprising absence of neuronal loss in the EC, the second layer of the EC, the amygdala, and MBC, and variable neuronal loss in all CA sectors, ranging from 0.9% in the CA2 to 30.5% in the CA1 and 15.5% in the subiculum. Equally surprising is the 25.8% loss of Purkinje cells not developing neurofibrillary degeneration, the 29.6% loss of neurons in the CN with mild neurofibrillary degeneration, and the 27.9% loss in the SN with neurofibrillary degeneration and β-amyloidosis.

### Ceiling level of the percentage of neuronal loss in 51- to 59-year-old DS subjects

The pattern of neuronal loss in moderate AD in 51- to 59-year-old DS subjects reflects a ceiling level of neuronal loss, essential for AD, in all layers of the EC (48.8%) and the second layer of the EC (69.5%), severe losses in all CA sectors (from 26.3% in CA4 to 30.1% in CA1 and 32.3% in CA2), and 26.7% loss in the subiculum, paralleled with a 27.9% loss in the amygdala and a 21.5% loss in the MBC (Table 7, Fig. 6).

**Table 7:** Estimated AD-associated neuronal loss in 51-to 59-y-old DS subjects (loss per day and per million neurons/day)

Structures/ subdivisions	Number of neurons in 43- to 49-y-old DS subjects, N = 4, Mean and (SD)	Number of neurons in 51-to 59-yr-old DS subjects, N = 17, Mean and (SD)	Neurons lost. difference between 43- to 49 and 51- to 59-y-old DS subjects	Number of neurons in DS reference group 26-to 41-y-old (100%)	Neurons lost (%) in 51–59-y-old DS subjects reference group, 26-to 41-y-old	*P* <	Number of neurons lost/day	Number of neurons lost/ million/day
Entorhinal c.all layers	10,470,333 (1,003,992)	5,702,533 (2,101,830)	4,767,800	9,779,100	48.8	0.002***	1520.8	145.3
Entorhinal c. islands	609,481 (68,719)	192,314 (84,474)	417,167	600,288	69.5	0.001***	133.1	218.3
CA1	5,271,466 (699,001)	2,986,260 (1,727,048)	2,285,206	7,584,266	30.1	0.05*	728.9	138.3
CA2	1,363,407 (350,731)	919,574 (287,449)	443,833	1,375,511	32.3	0.04*	141.6	103.8
CA3	969,837 (225,781)	655,428 (255,405)	314,409	1,062,288	29.6	ns	100.3	103.4
CA4	950,304 (255,206)	658,923 (467,298)	291,381	1,105,866	26.3	ns	92.9	97.8
Subiculum	2,575,933 (293,733)	1,760,757 (562,251)	815,176	3,049,650	26.7	0.03*	260.0	100.9
Amygdala	7,150,380 (940,312)	5,203,993 (1,311,409)	1,946,387	6,978,730	27.9	0.02*	620.9	86.8
Thalamus	13,488,304 (1,821,914)	12,891,781 (2,374,425)	596,524	14,774,176	4.0	ns	190.3	14.1
Substantia nigra	396,519 (76,289)	309,905 (65,494)	86,614	550,053	15.7	0.05*	27.6	69.7
MBC	994,389 (104,089)	783,233 (205,770)	211,156	981,577	21.5	ns	67.4	67.7
Caudate n	29,151,928 (7,214,428)	27,913,089 (10,600,000)	1,238,840	41,434,112	3.0	ns	395.2	13.6
Purkinje cells	10,569,930 (720,445)	11,715,680 (2,207,151)	0	14,247,128				
Dentate n	463,150 (138,022)	520,173 (96,537)	0	510,829				
			Total loss: 13,414,493 neurons		Mean loss: 28.0%		Mean loss: 356.6/d Total loss: 4,278.9/d	Mean loss: 96.6/ million/d

Loss calculated using the 8.6-year (3,135-day) difference in mean age between the groups as the denominator. *p < 0.05; **p < 0.01; ***p < 0.001

### Decline of neuronal loss in 61- to 72-year-old DS subjects with prevalence of severe dementia

A 4.6 × and 15.7 × decline of neuronal loss in the EC and the second layer of the EC, respectively, a 14.6 × decline in the CA2 and reduction of neuronal loss from 29.6% in CA3 in 5th decade to 0 in sixth decade, and a 28 × decline in the amygdala in the 5th and 6th decades reflect depletion of the pool of neurons during the 16 years of mild and moderate AD in DS subjects 43–59 years of age. However, although the majority of examined structures reveal a floor level in the 61–72-year-old group (Table 8, Fig. 6), the thalamus shows a local increase in neuronal loss from 4.03% to 22.0%, whereas the MBC shows a plateau with 21.5% and 22.7% loss in the 5th and 6th decades respectively, reflecting structure-specific trajectories of AD neuronal loss in the DS cohort.

**Table 8:** Estimated AD-associated neuronal loss in 61–72-y-old DS subjects (loss per day and per million neurons/day)

Structures/ subdivisions	Number of neurons in 51- to 59-y-old DS subjects, N = 17, Mean (SD)	Number of neurons in 61-to 72-y-old DS subjects, N = 8 Mean (SD)	Neurons lost. Difference between 51-to 59 and 61- to 72-y-old DS subject	Number of neurons in DS reference group 26-to 41-y-old (100%) DS subjects	Neurons lost (%) in 61–72-y-old DS subjects. Reference group (100%) 26-to 41-y-old	*P* <	Number of neurons lost/day	Number of neurons lost/ million/day
Entorhinal c. -all layers	5,702,533 (2,101,830)	4,666,150 (1,533,640)	1,036,383	9,779,100	10.6	ns	282.9	49.6
Entorhinal c. -islands	192,314 (84,474)	165,633 (68,200)	26,681	600,288	4.4	ns	7.3	37.9
CA1	2,986,260 (1,727,048)	2,215,244 (1,185,499)	771,016	7,584,266	10.2	ns	210.5	70.5
CA2	919,574 (287,449)	889,200 (203,703)	30,374	1,375,511	2.2	ns	8.3	9.0
CA3	655,428 (255,405)	720,055 (201,407)	0	1,062,288				
CA4	658,923 (467,298)	541,710 (202,098)	117,213	1,105,866	10.6	ns	32.0	48.6
Subiculum	1,760,757 (562,251)	1,524,200 (385,960)	236,557	3,049,650	7.8	ns	64.6	36.7
Amygdala	5,203,993 (1,311,409)	5,340,980 (1,023,388)	0	6,978,730				
Thalamus	12,891,781 (2,374,425)	9,635,097 (2,965,348)	3,256,684	14,774,176	22.0	0.02*	889.1	69.0
Substantia nigra	309,905 (65,494)	265,558 (44,657)	44,347	550,053	8.1	ns	12.1	39.1
MBC	783,233 (205,770)	560,206 (200,686)	223,027	981,577	22.7	0.04*	60.9	77.7
Caudate n	27,913,089 (10,600,000)	28,785,213 (5,986,303)	0	41,434,112				
Purkinje cells	11,715,680 (2,207,151)	9,790,558 (4,117,606)	1,925,122	14,247,128	13.5	ns	525.6	44.9
Dentate n	520,173 (96,537)	473,472 (100,863)	46,701	510,829	9.1	ns	12.7	24.5
			Total loss: 7,714,105 neurons		Mean loss: 11.0%		Mean loss: 191.5/d Total loss: 2,106.0 /d	Mean loss: 46.1/ million/d

^*^Calculated using the 10.04-year (3,663-day) difference in mean age between the groups as the denominator. AD-associated neuronal loss (%) calculated in all age groups in reference to baseline number of neurons in DS subjects 26–41 years of age with developmental deficit but no or minimal pre-AD pathology

The total regional cumulative neuronal loss (%) in 43- to 72-year-old DS/AD subjects revealed, as was expected for this dementing disorder, the most severe total neuronal loss in the EC (59.4%) and the second layer of the EC (73.9%) as well as in all CA sectors, ranging from 70.8% in CA1 to 35.4% in CA2, and 50% loss in the subiculum. These losses were paralleled by prominent neuronal losses in the dopaminergic (SN, 51.7%) and cholinergic (MBC, 44.2%) systems as well as in the amygdala (27.9%) and thalamus (34.7%) (Fig. 6).

The general pattern of neuronal loss in three age stages of AD in DS subjects is reflected in an 11.9% mean neuronal loss in all 14 structures in the 4th decade, a two-fold increase in mean neuronal loss to 28.0% in the 5th decade, but an almost 2.7-fold decrease to 11.0% in the sixth decade with its prevalence of severe AD. The global relationships between the magnitude of developmental neuronal deficits and AD-associated neuronal loss are characterized by a very similar total developmental neuronal deficit of 40.8 million and an AD-associated total neuronal loss in 43- to 72-year-old DS subjects estimated as 41.6 million (Fig. 6**).**

### Staging of dynamics of neuronal loss–number of neurons lost per day and normalized rate of neuronal loss per million neurons/day

**Fig. 7:**
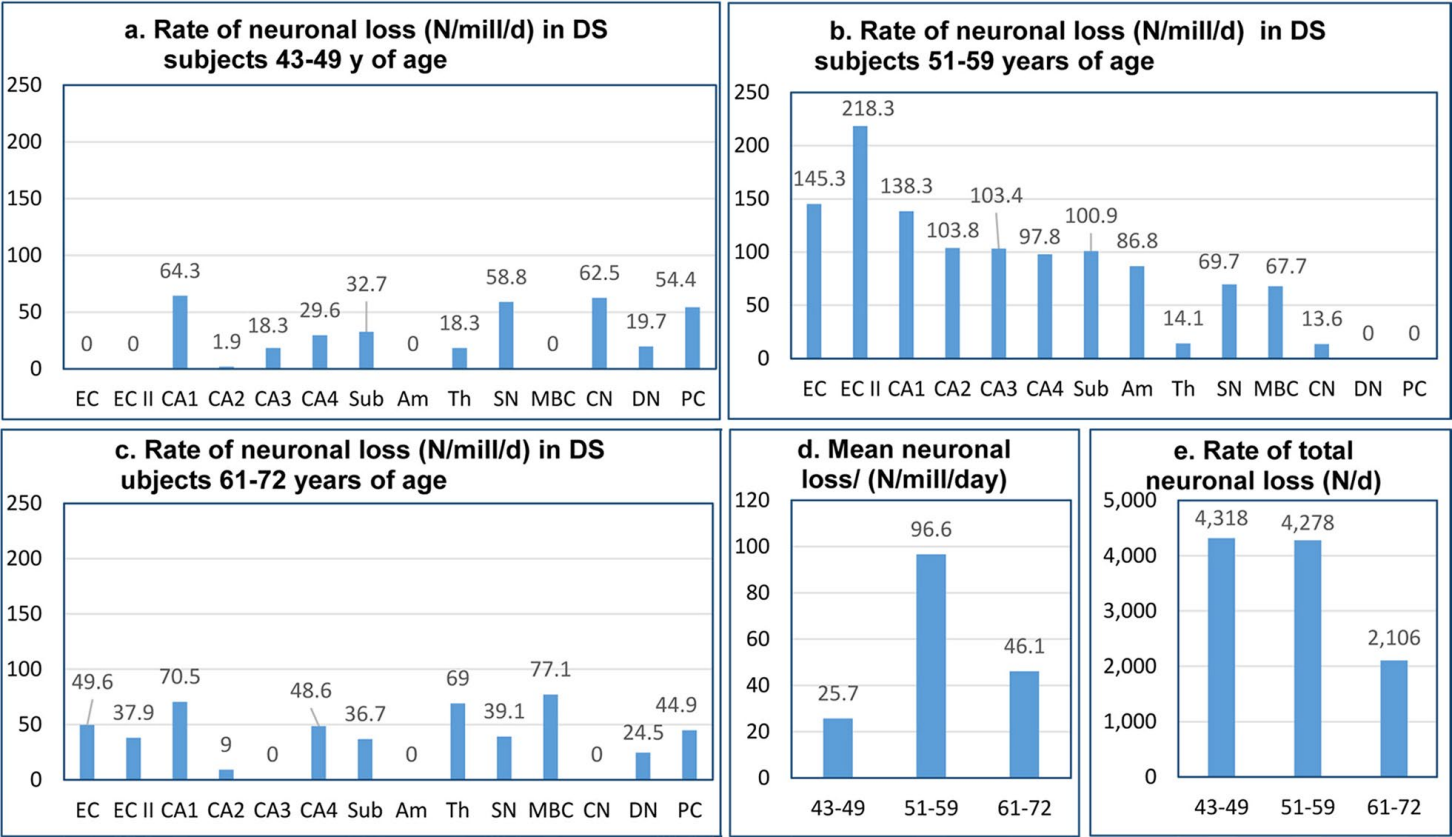
Rate of neuronal loss. The speed of neuronal loss is defined by the estimated rate of neuronal loss per day and the number of lost neurons per million neurons/day. Graphs **a-c** illustrate regional differences in the rate of neuronal loss in the fourth, fifth, and sixth decades of life, respectively, of DS subjects. **b** and** d** reveal the top level of the rate of neuronal loss in the fifth decade. The cumulative rate of neuronal loss (number of lost neurons per day in all examined regions) is similar in the fourth and fifth decades (4,318/day and 4,278/day, respectively) but decreases by half in the sixth decade (2,106/day) (**e**)

The index of neuronal loss calculated per million neurons per day is independent of the size of the neuronal population in the examined structure and provides comparable measures of the rate of neuronal loss. The rate of neuronal loss in DS subjects 43–49 years of age is relatively low and varies broadly, from undetectable in the EC and EC second layer, the amygdala, and MBC to a loss of 64.3 neurons/million/day in the CA1, 58.8 in the SN, 62.5 in the CN, and loss of 54.4 Purkinje cells/million/day (Table 6, Fig. 7). The highest rate of neuronal loss is detected in 51- to 59-year-old DS subjects, with an especially high rate in the EC (145.3), the second layer of the EC (218.3), and the CA, ranging from 138.3 in CA1 to 97.8/million/day in CA4 (Table 7, Fig. 7). However, the rate of neuronal loss decreases several-fold in 61- to 72-year-old DS subjects (Table 8, Fig. 7).

The global pattern of dynamics of neuronal loss is reflected in an increase in the mean rate of neuronal loss in examined regions from 25.7 neurons/million/day in the fourth decade to 96.6 neurons/million/day in the fifth decade, but a decrease in the late stage of AD to 46.1 neurons/million/day in the sixth decade of life of DS subjects. The direct index of total neuronal loss (N/day) in examined structures, declining from 4,318/day in the fourth decade, to 4,278 in the fifth decade and 2,106 in the sixth decade, reflects a diminishing pool of neurons in examined structures and a diminished speed of neuronal loss (Fig. 7).

### Staging of neurofibrillary degeneration in DS/AD

**Fig. 8:**
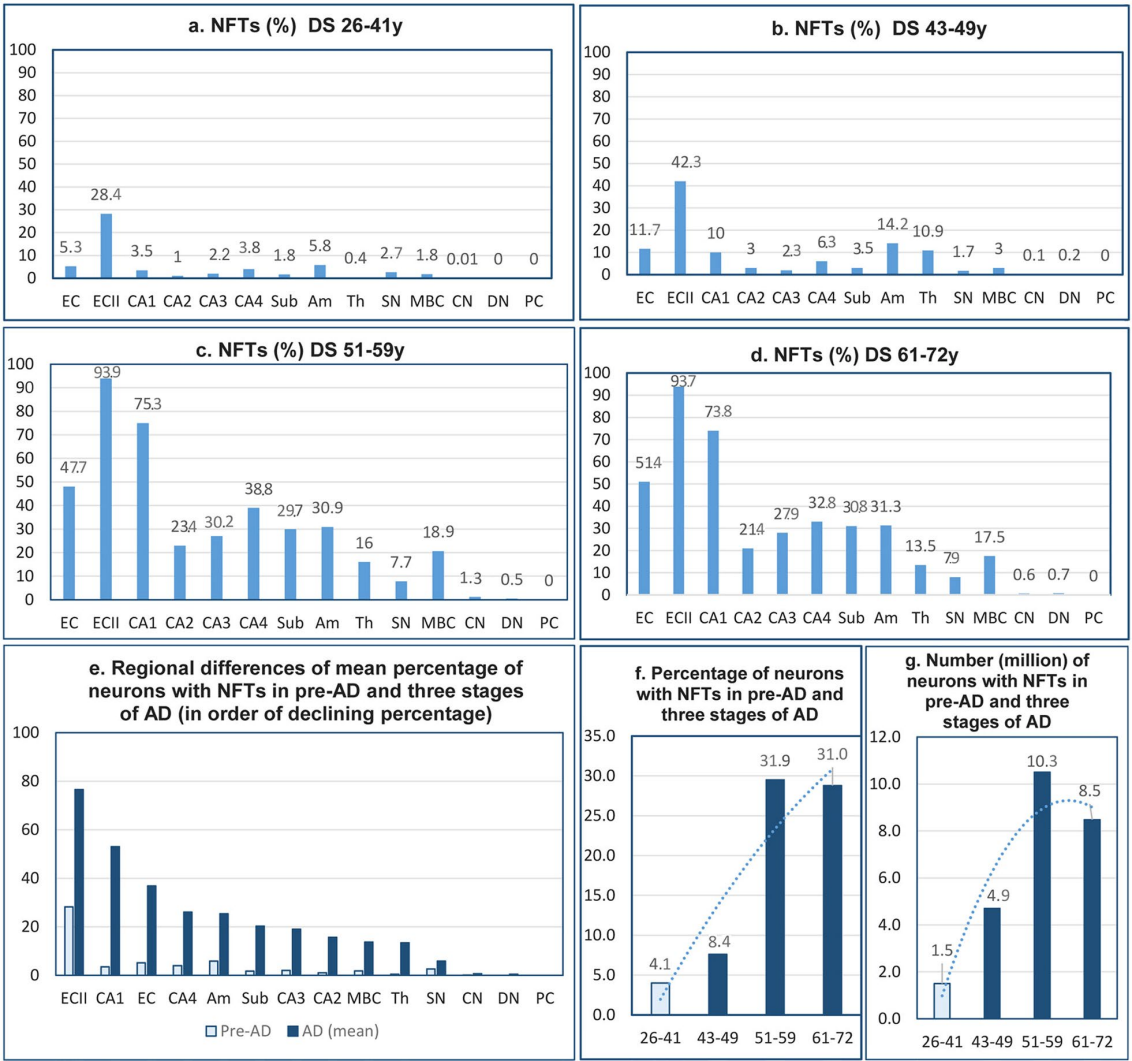
Neurofibrillary degeneration. Graphs **a-e** illustrate regional differences in the percentage of neurons with NFTs and the progression of neurofibrillary degeneration from the incipient stage in pre-AD (26- to 41-year-old DS subjects) to three stages of AD in the fourth, fifth, and sixth decades. Fig. **e** shows the ratio between the percentage of neurons with NFTs in pre-AD and in AD and the gradient of susceptibility of neurons, with the highest in the entorhinal cortex, cornu Ammonis, subiculum, and amygdala; lower in the MBC, thalamus, and substantia nigra; and marginal in the caudate and dentate nuclei. The general pattern is illustrated in fig. **f** and **g**, showing a plateau of the percentage of neurons with NFTs in the fifth and sixth decades. Decrease of the number of neurons with NFTs from 10.3 million in the fifth decade to 8.5 million in the sixth decade is evidence of the death and removal of neurons with NFTs

In the pre-AD stage in 26- to 41-year-old DS subjects, neurons with NFTs were detected in 12 examined structures, but not in the dentate nucleus and Purkinje cells. The mean percentage of NFT-positive neurons in this group was 4.1%, but the percentage of neurons with NFTs was relatively high in the second layer of the EC (28.4%) and in the amygdala (5.8%) (Table 9, Fig. 8). The mean percentage of NFT-positive neurons increased to 7.6% in 43- to 49-year-old DS subjects with mild AD. The result of further enhancement of neurofibrillary degeneration in 51-to 59-year-old DS subjects was a several-fold increase in the percentage of neurons with NFTs to a mean of 29.5%. The most prominent increase of degeneration was detected in the second layer of the EC (94%), all layers of the EC (48%), and CA1 (75%). Approximately 20–30% of neurons were affected by neurofibrillary degeneration in other segments of the CA, subiculum, amygdala, and MBC. A sign of a plateau of neurofibrillary degeneration was the stabilization of a similar percentage of neurons with NFTs (mean 28.8%) in DS subjects 61–72 years of age with severe AD, but with a very high percentage of neurons with NFTs in the second layer of the EC (94%), all layers of the EC (51%), and CA1 (74%). The total number of neurons with NFTs in all examined structures increased from 1.6 million in 26- to 41-year-old DS subjects in the pre-AD stage to 4.7 million in the fourth decade, and a ceiling level in the fifth decade, with 10.5 million neurons with NFTs. The reduction in the pool of brain neurons and the death of neurons with NFTs result in a decrease in the number of NFTs to 8.5 million in 61- to 72-year-old DS subjects.

**Table 9:** Brain-region and neuron-type specific number and percentage of neurons with NFTs in four DS age groups

Structures/ subdivisions	**DS 26-41y**			**DS 43–49**			**DS 51–59**			**DS 61–72**		
	Number	Std.dev	%	Number	Std.dev	%	Number	Std.dev	%	Number	Std.dev	%
Entorhinal cortex**—** all layers	514,085	489,465	5.3	1268,542	831,686	11.7	2,677,856	1,037,522	47.7	2,231,773	579,014	51.4
Entorhinal cortex**—**islands	146,574	162,300	28.4	256,579	109,260	42.3	179,363	75,840	93.9	156,215	67,649	93.7
CA1	223,781	236,938	3.5	569,738	589,164	10.0	2,194,866	1,311,918	75.3	1,466,271	601,308	73.8
CA2	13,140	17,748	1.0	41,525	22,381	3.0	194,360	83,202	23.4	177,765	100,826	21.4
CA3	15,185	17,566	2.2	25,010	22,499	2.3	177,270	71,494	30.2	182,371	106,649	27.9
CA4	36,758	43,532	3.8	63,735	26,396	6.3	218,396	131,984	38.8	164,995	81,514	32.8
Subiculum	47,304	54,955	1.8	92,647	50,976	3.5	522,375	276,322	29.7	468,360	153,094	30.8
Amygdala	394,698	336,152	5.8	1,114,323	1,402,649	14.2	1,558,170	454,192	30.9	1,628,256	482,296	31.3
Thalamus	57,382	50,262	0.4	1,456,602	1,055,296	10.9	2,069,313	858,102	16.0	1,813,647	664,388	13.5
Substantia nigra	16,719	28,958	2.7	5,246	10,492	1.7	23,633	13,163	7.7	20,880	5,545	7.9
MBC	19,178	33,040	1.8	31,660	24,200	3.0	132,521	68,454	18.9	98,998	81,179	17.5
Caudate nucleus	4,006	8,012	0.01	18,038	36,075	0.1	327,071	241,425	1.3	142,340	123,159	0.6
Purkinje cells	0	0	0	0	0	0	0	0	0	0	0	0
Dentate nucleus	0	0	0	1,323	1,603	0.2	2,588	1,432	0.5	3,363	1,670	0.7
Total number; mean %	1,488,810		4.1	4,944,968		8.4	10,277,782		31.9	8,555,234		31.0

### Staging of β-amyloidosis in DS/AD

**Fig. 9:**
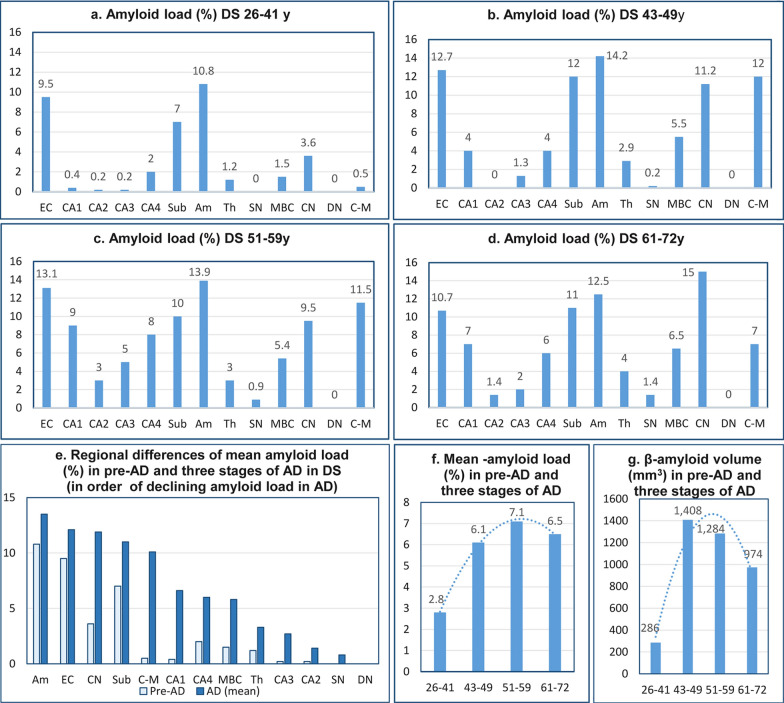
Amyloid load (%). Fig. **a** shows the onset of amyloidosis in pre-AD with marked amyloid load in the entorhinal cortex, amygdala, and subiculum, and much less in the CN, CA4, MBC, and thalamus. Amyloid load increases in a region-specific range and stabilizes to comparable levels in the fourth, fifth, and sixth decades **(b-d,** respectively**)**. Graph **e** illustrates grading of regional susceptibility to β-amyloidosis and shows that the pattern in pre-AD predicts severity of regional amyloidosis in next decades. Fig. **f** and **g** show a global pattern of onset and progression of amyloidosis in pre-AD and in the fourth and fifth decades but a decrease of amyloid load (%) and amyloid volume (mm^3^) in the sixth decade of life of DS subjects

Amyloidosis shows significant regional differences, but regional patterns of high or low Aβ load are preserved during the entire course of the disease (Table 10, Fig. 9a-e). High mean amyloid load in the amygdala, entorhinal cortex, and subiculum (10.8%, 9.5%, and 7.0%, respectively) in the pre-AD stage appears to be a predictor of high (13.5%, 12.2%, and 11.0%, respectively) mean amyloid load during 30 years of AD. The mean amyloid load increases from 2.8% in pre-AD to 6.1%, 7.1%, and 6.5% in the fourth, fifth, and sixth decades of the DS/AD subject’s life (Fig. 9f). Direct estimates of amyloid deposits (mm^3^) reveal that the transition from the pre-AD stage (26–41 y of age) (286 mm^3^) to the fourth decade (43–49 y) results in an almost five-fold increase of amyloid volume to 1,408 mm^3^. The decrease of total volume of amyloid-β deposits in the fifth and sixth decades to 1,284 mm^3^ and 974 mm^3^ (Fig. 9g) respectively reflects the consequences of early-onset AD-associated loss of neurons and exhaustion of this main source of Aβ, resulting in a decline of the amyloid pool in plaques and diffuse deposits.

**Table 10:** Brain region–specific amyloid volume (mm^3^) and amyloid load (%) in four DS age groups

Structures/ subdivisions	DS 26–41 y		DS 43–49 y		DS 51–59 y		DS 61–72 y	
	Volume	Load	Volume	Load	Volume	Load	Volume	Load
Entorhinal cortex—all layers	58.2	9.5	77.7	12.7	56.4	13.1	38.8	10.7
CA1	1.3	0.4	10.3	4.0	18.4	9.0	10.4	7.0
CA2	0.1	0.2	0.0	0.0	1.4	3.0	0.4	1.4
CA3	0.1	0.2	0.4	1.3	1.6	5.0	0.7	2.0
CA4	1.3	2.0	2.2	4.0	5.6	8.0	3.3	6.0
Subiculum	10.9	7.0	16.9	12.0	10.0	10.0	8.6	11.0
Amygdala	45.4	10.8	61.6	14.2	54.3	13.9	40.4	12.5
Thalamus	36.0	1.2	74.6	2.9	74.2	3.0	80.6	4.0
Substantia nigra	0.0	0.0	0.2	0.2	0.8	0.9	1.0	1.4
MBC	2.3	1.5	9.4	5.5	7.3	5.4	14.4	6.5
Caudate nucleus	81.8	3.6	189.9	11.2	140.0	9.5	222.7	15.0
Cerebellar cortex molecular layer	49.1	0.5	965.5	12.0	914.5	11.5	561.3	7.0
Dentate nucleus	0.0	0.0	0.0	0.0	0.0	0.0	0.0	0.0
Total (sum) Vol and mean load	286.5	2.8	1,408.6	6.1	1,284.5	7.1	974.3	6.5

### Correlations between neuron number and structure volume decline and the number and percentage of neurons with NFTs

Strong correlations in almost all examined structures between neuron number and the percentage of neurons with NFTs reflect the critical role of neurofibrillary degeneration in AD neuronal loss and functional decline. Correlations between declining neuron number and the volumes of all examined structures mirror the leading role of neurofibrillary degeneration and neuronal loss in brain atrophy (Table 11). The pattern detected is consistent with clinicopathological staging of the dynamics of pathological changes with the following ceiling levels: total volume loss (2,953 mm^3^) in the fourth decade, mean percentage of volume loss (15.7%) in the fifth decade, total number of lost neurons (20.4 million) in the fourth decade, mean percentage of neuronal loss (28.0%) in the fifth decade, mean rate of neuronal loss (96.6 million/day) in the fifth decade, top level of the rate of neuronal loss in the fourth/fifth decades (4,318/day and 4,278/day, respectively), highest level of the percentage of neurons with NFTs in the fifth decade (29.5%/28.8%, respectively), highest level of the number of neurons with NFTs (10.5 million) in the fifth decade, highest level of mean amyloid load (7.1%) in the fifth decade, and highest level of amyloid volume (1,408 mm3) in the fourth decade.

**Table 11:** Correlations in DS cohort

Structure	Correlation with neuron number of:		
	Structure volume	NFT number	NFT%
Entorhinal cortex—all layers	0.89***	−0.09	− 0.74***
Entorhinal cortex —islands	0.93***	0.25	− 0.89***
CA1	0.92***	− 0.01	− 0.76***
CA2	0.79***	− 0.44^*^	− 0.66***
CA3	0.80***	− 0.24	− 0.61***
CA4	0.41*	− 0.29	− 0.62***
Subiculum	0.92***	− 0.17	− 0.53***
Amygdala	0.60***	0.04	− 0.46***
Thalamus	0.53^***^	− 0.31	− 0.37
Substantia nigra	0.85***	− 0.06	− 0.41*
Magnocellular basal complex	0.85***	− 0.16	− 0.50**
CN	0.87***	− 0.20	− 0.38*
DN	0.22	0.28	− 0.15

*p < 0.05; **p < 0.01; ***p < 0.001

### Enhancement of amyloid angiopathy in the EC, CA1, subiculum, and molecular layer of the cerebellar cortex in the sixth decade of life of DS/AD subjects

Amyloid angiopathy was detected in all four regions in the wall of capillaries as well as in the tunica media in the wall of arterioles and small-caliber arteries in all examined DS subjects. The numerical density of amyloid-positive vessels ranged from 3.19/mm^2^ in the EC to 6.88/mm^2^ in the molecular layer of the cerebellar cortex in 61- to 72-year-old DS subjects. The study revealed age-associated advancement of amyloid angiopathy reflected in the higher number of positive vessels in 61- to 72-year-old DS/AD subjects in comparison to 41- to 59-year-old DS/AD subjects by 30.8% in the EC, 21.4% in CA1, 49.9% in the subiculum, and 21.0% in the molecular layer of the cerebellar cortex.

### Prevalence and severity of Lewy body degeneration in the amygdala in DS/AD subjects

The immunostaining with mouse mAb 4B12 detecting α-synuclein in Lewy bodies in neurons in the amygdala in 12 of 22 (54.5%) DS subjects 41–59 years of age and in six of eight examined subjects 61–72 years of age (75%) reflects the age-associated increase in the prevalence of α-synucleinopathy in DS. In the amygdala of DS subjects, the estimated numerical density of Lewy bodies was 761/mm^3^, and the average total number per case was 290,647, with 4.6% of neurons being Lewy body positive. However, the decline in the number of neurons with Lewy bodies from 861/mm^3^ to 575/mm^3^ (−33%), and in the total number of neurons with Lewy bodies from 337,000 to 201,000 in 61- to 72-year-old DS subjects indicates that α-synucleinopathy results in neuronal death in DS/AD subjects and in removal of 39.9% of affected neurons in the amygdala.

### Low prevalence of TDP-43 neurodegeneration and percentage of affected neurons in DS cohort

Immunostaining with rabbit polyclonal phosphorylation–independent anti-TDP-43 antibody 10,782–1-AP revealed cytoplasmic inclusions of aggregated TDP-43 protein in the amygdala of only three of 33 DS subjects (9.0%), who died at 59, 61, and 65 years of age. The percentage of neurons with cytoplasmic TDP-43–positive inclusions was 0.8, 0.4, and 7.9, respectively, and the estimated numerical density of positive neurons was 88/mm^3^, 53/mm^3^, and 1,452/mm^3^), respectively.

### The pattern of major age-associated pathological processes in DS/AD cohort

**Fig. 10:**
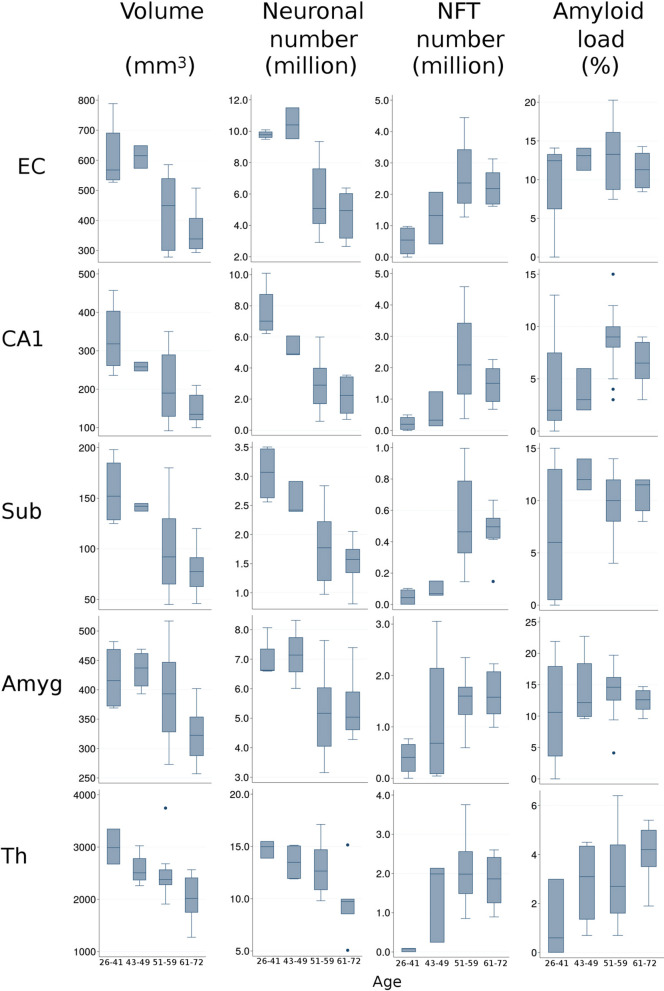
Overview of the staging of AD-associated changes of examined structures volume (mm^3^), number of neurons and NFTs (million), and amyloid load (%) in the entorhinal cortex (EC), cornu Ammonis sector 1 (CA1), subiculum (Sub), amygdala (Amyg), and thalamus (Th) in DS subjects 26–41, 43–49, 51–59, and 61–72 years of age. Graphs illustrate structure-specific staging of brain structures volume and neuronal number decline paralleled with the growth of the number of neurons with NFTs and increase of amyloid load associated with functional decline, including onset and progression of dementia in the fifth/sixth decades of life of subjects with trisomy 21. The upper and lower boundaries of the box represent interquartile range (IQR). The mean value is marked with the horizontal line within the box. The “whiskers” mark the maximal and minimal values unless any data point lies more than 1.5 times the IQR above the 75th percentile or below the 25th percentile. Outliers are marked with dots

Figure 10 provides overview of onset of neurofibrillary degeneration in 26–41-year-old DS subjects, two decades of progress of neurodegeneration culminating at an age of around 60 years and decline in the sixth decade. Neurofibrillary degeneration results in three decades of neuronal loss, atrophy of examined structures and functional deterioration including dementia. The increase of amyloid load most prominent in 26–41-year-old individuals is less intense in the next three decades and decreases in the 6th decade in response to exhaustion of Aβ supply by severely downsized neuronal population.

## Discussion

### Trisomy 21–associated structural developmental deficits in the brain of DS subjects—the baseline for estimation of early-onset AD pathology in DS

This and other studies demonstrate that the reduced size of brain structures in adults with DS is a combined effect of developmental deficits and acquired AD pathology [16] and indicate that estimation of AD-associated neuronal loss in older DS subjects must be calculated as the percentage of the number of neurons in AD pathology–free young DS subjects [48]. In DS, neurodevelopmental pathologies, including deficits of neurogenesis, synaptogenesis, and lamination, contribute to the reduced size of brain structures and the reduced number of neurons and synapses [72, 103, 106]. This study of 14 brain regions characterized AD-related topography and severity of volume loss and loss of neurons in the context of trisomy 21–related topography and severity of developmental brain volume and number of neuron deficits.

### Region-specific developmental brain volume deficits in DS

This study of DS subjects’ brains in the pre-AD stage of life (26–41 years of age) revealed a 12.8% mean developmental volume deficit in 14 structures and regional volume deficits by 27.2% in CA1, 36.2% in CA4, 17% in ECII, 14.9% in the subiculum, and 7.0% in the amygdala. A similar range of developmental changes was detected in the cerebellum, with a 31.6% deficit in the volume of the molecular layer of the cerebellar cortex and a 23.1% volume deficit in the dentate nucleus. Deficits distinguished in 11 structures and the absence of developmental deficits in the CA2, substantia nigra, and MBC reflect regional differences in defective brain development and implicate their different contributions to functional developmental deficits in individuals with DS.

These observations are consistent with results of MRI studies of the brains of fetuses [60, 82], infants and toddlers [78], and teenagers [63] with trisomy 21. Fetal and neonatal MRI assessment of developmental brain alterations indicates that brain pathology in DS originates in utero and continues over the lifespan. Fetuses with DS had decreased cerebellar hemispheric (*p* < 0.0001), whole cerebellum (*p* = 0.043), cortical plate (*p* = 0.033), and subcortical parenchymal volume (*p* = 0.010) compared to control fetuses. Such differences began at around GW 28 and increased during gestation [82]. The reduced cerebellar volume detected in the second trimester and the significant reduction of cortical volume evident during the third trimester are considered the substrate for neurocognitive impairment [60]. MRI-based studies show reflections of fetal pathology in infants and toddlers as the deficit of cerebellar gray matter [78] as well as smaller brain, cerebellum, and hippocampus volume in 5- to 23-year-old DS subjects [62, 63]. According to Pinter et al. [62], the stable nature of these abnormalities may reflect their contribution to the cognitive and functional deficits in DS subjects.

### Region-specific developmental neuronal deficits in DS

Widespread hypoplasia in the brain of 16- to 21-week-old fetuses with karyotypically proven trisomy 21 is associated with deficits of brain structure volume and number of neurons, including deficits of 39% in the total number of granule cells in the dentate gyrus, of 35% in neurons in the hippocampal pyramidal layer, of 38% in neurons in the presubiculum lamina principalis interna, and of 32% in neurons in the lamina principalis externa. In the EC, neuronal deficit ranges between 32% in the lamina principalis interna and 37% in the lamina principalis externa [24]. Comparison by Larsen et al. [41] of the total cell number in the major fetal brain regions, including the cortical plate, the subplate, and the intermediate and ventricular zones, revealed that the total cell number in 19-week-old fetuses with trisomy 21 (6.85 billion) is 34% less than the total cell number in age-matched control fetuses (10.4 billion). The reduced neuron number detected in DS fetuses and children [11, 24, 41] indicates that trisomy-related developmental abnormalities of neurogenesis are a major contributor to multiregional hypoplasia in the brains of DS individuals and to functional deficits including intellectual deficits [41].

### Comparable magnitude of regional developmental neuronal deficits in pre-AD DS subjects and magnitude of regional losses of neuronal reserve in aging: the baseline for onset of AD pathology in DS and in sAD

The baseline for EOAD in subjects with trisomy 21 and DS is defined by brain region–specific neuronal developmental deficits and enhanced apoptotic death [11, 24], which are reflected in developmental deficits of 18.4% of neurons in the EC, 37.7% in the second layer of the EC, 38.6% in the CA1, 50.8% in the amygdala, 52.5% in the thalamus, and 66.5% in the dentate nucleus, and a 34.8% deficit of Purkinje cells detected in this study. This pattern of developmental region–specific volume and neuronal deficits supports the concept of Annus et al. [3] that the DS brain is not simply a downscaled model of the typically developed brain but is a trisomy-specific product of distinct types, topography, and severity of developmental changes resulting in disproportions between differently deficient regions and loss of structural and functional integrity.

In a nondemented control cohort, the baseline for LOAD is defined by a loss of neurons without signs of functional decline (neuronal reserve). This loss was determined in neurologically typical subjects 25–102 years of age to be an age-associated loss of 33.3% of neurons in the EC, 54% in the second layer of the EC, 28.5% in the CA1, 45.8% in the amygdala, 40.5% in the thalamus, and 40.1% in the dentate nucleus as well as a 48.5% loss of Purkinje cells [93]. Age-associated loss of neuronal reserve and remodeling of neuronal networks might be considered the baseline for estimation and staging of an almost 20-year-long process of brain-region–specific AD neurodegeneration and neuronal loss resulting in the onset and progression of AD dementia.

Despite topographic and numerical similarities, two fundamentally different processes reflecting abnormal brain development in trisomic DS subjects and age-associated brain remodeling leading to loss of neuronal reserve in the control cohort independently lower the threshold of onset of symptomatic AD pathology and functional decline with dementia in EOAD in the DS cohort and dementia in LOAD in the general population. The magnitude of these changes is reflected in the neuronal deficit of 40.8 million in 14 examined structures in 26- to 41-year-old DS subjects and a very similar AD-associated 41.6 million total loss of neurons contributing to functional decline including dementia in 43- to 72-year-old DS/AD subjects.

### Correlation between age as well as stage of dementia and decline of neuron number in DS/AD

The study shows that the effect of trisomy 21 is early onset of AD pathology in the DS cohort, strong correlations between age and decline of the number of neurons in the entorhinal cortex, sectors of the cornu Ammonis, amygdala, thalamus, substantia nigra, magnocellular basal complex, associated with early onset of dementia and strong correlation between stage of dementia and AD pathology including reduction of the number of neurons and examined structure volume. Very strong correlations between both age and stage of dementia and structural changes detected in the examined cohort are consistent with a 23% prevalence of dementia at age of 50 years, 45% at age 55 years, and 88% or more at 65 years [53, 64, 108] and the results of Ballard et al. [5] showing the prevalence of progressive cognitive decline in 55% of DS patients 40–49 years and 77% in patients 60–69 years of age. Detection of correlations between structural changes and both age and stage of dementia integrates three components of AD pathology in DS and suggests that successful preventive treatment before the age of 50 may disrupt/block this highly predictable cascade of pathology including neuronal loss and loss of function. In contrast to the genetically regulated, highly predictable age of onset and progression of the cascade of AD structural and functional changes in DS, the broad range of age of onset of AD pathology and dementia in sporadic AD [7] results in a lack of correlation between age and severity of AD pathology in LOAD [92].

### Different dynamics of volume and neuronal loss in sporadic LOAD and EOAD in DS cohort

Estimates of AD-associated mean volume losses show a significantly different pattern in these two cohorts, with an increase in mean volume loss in LOAD FAST 3–4, 5–6, and 7 from 22.2% to 28.3% and 43.6%, respectively, whereas mean volume losses in EOAD in developmentally deficient DS brains were less and were relatively stable in the age groups of 43- to 49-, 51- to 59-, and 61- to 72-year-old DS subjects, with 12.7%, 15.7%, and 10.1% loss, respectively.

The mean rate of neuronal loss (mean number of neurons lost per million neurons/day) shows another striking difference in stage-specific dynamics of AD pathology in these two cohorts, with moderate neuronal loss in 43- to 49-year-old DS subjects (25.7/million/day) but more than a six-fold higher rate of neuronal loss (159 neurons/million/day) in FAST 3–4 in sAD, and a ceiling level of neuronal loss (96.6/million/day) in 51- to 59-year-old DS subjects, but a 3.4 times higher loss (332/million/day) in corresponding FAST 5–6 in sporadic AD. However, the decrease in the rate of neuronal loss to a floor level (46.1/million/day) in 61- to 72-year-old DS subjects was relatively similar (64/million/day) to the floor level in FAST 7 in the sAD cohort.

Our previous study of a LOAD cohort revealed that the mean percentage of neuronal loss decreases from a ceiling level of 31.0% in MCI/mild AD (FAST 3–4) to 15.5% in moderate/moderately severe AD (FAST 5–6), and to 13.8% in severe AD (FAST 7) [94], whereas in this study of EOAD, in the DS cohort, the mean neuronal loss in three corresponding age groups is partly different, with a 11.9% loss in the fourth decade, an increase to a ceiling level of 28.0% in the fifth decade, and a decline to 11.0% in the sixth decade of life of DS subjects. Lower overall neuronal loss in older DS subjects than in subjects with advanced sAD was also reported by Mann et al. [48], and the difference was interpreted as related to a developmentally lower number of neurons in young DS subjects than in age-matched control individuals.

### Mechanisms of neuronal loss in DS cohort

Staging of progression of neurofibrillary degeneration revealed a comparable increase in the percentage of neurons with NFTs in each of three FAST stages in LOAD, from 13.0% to 24.0% and 31.0% [94], and a similar increase in three age groups of the DS/AD cohort, with an increase from 7.6% to 29.5% and 28.8%. This and other studies [6] reveal that late-life cognitive decline in AD is driven by a variety of age-related pathologies and suggest that interpretation of neuronal loss in the DS/AD cohort requires consideration of the role of neurofibrillary degeneration as well as other proteinopathies including α-synucleinopathy with Lewy bodies, TDP43 protein aggregation, β-amyloidosis, and amyloid angiopathy. This broader range of data may be helpful in the design of multi-target treatments and of the timing of treatments matching the clinicopathological staging of AD and DS/AD that can disrupt efficiently the chain of synergistic interactions leading to escalation of mutually related pathomechanisms, neuronal loss, and functional decline including dementia.

### Low prevalence and limited percentage of neurons with TDP-43–positive cytoplasmic inclusions in DS/AD subjects

To evaluate the potential contribution of other proteinopathies to neuronal degeneration and loss, as well as the loss of function in the DS cohort, the prevalence (number and percentage of cases with TDP-43 degeneration) and the severity of this degeneration (percentage of neurons with intracellular TDP-43–positive inclusions) were determined in the amygdala. Hyperphosphorylated transactive response DNA-binding protein 43 (TDP-43) is a nuclear ribonucleo-protein involved in protein processing and exon splicing [46, 89]. In pathological conditions including AD and limbic-predominant age-related TDP-43 encephalopathy (LATE), abnormally phosphorylated TDP-43 is aggregated in the cell nucleus and in multiple cytoplasmic inclusions. In sAD and DS/AD, the most affected are neurons in the amygdala, adjacent EC, and hippocampus (dentate gyrus) [4, 46, 58]. TDP-43 neurodegeneration extended from the amygdala to other brain structures is frequently associated with increased clinical expression of AD dementia [32, 35] and is considered an integral component of at least some forms of AD [17].

The low prevalence of TDP-43 degeneration—14% [14], (8.9% [15], and 9%—detected in this study distinguishes subjects with DS from four populations with sporadic AD, with 23% [1], 26% [87], 58% [38], and 56% [4] positive cases. Our clinicopathological staging of sAD revealed TDP-43 degeneration in only 1 of 11 AD cases diagnosed with early stages of AD (FAST 3–5), but the prevalence increased to 68.7% in moderately severe and severe AD (FAST 6–7) [94]. The absence of TDP-43 pathology in a substantial percentage of AD cases [1, 85] is considered an indication that TDP-43 neurodegeneration is not essential for developing AD [85, 86]. However, TDP-43 pathology still may modulate AD pathology and affect the clinical course of disease [8, 33, 59]. The low prevalence of TDP-43 pathology in the examined DS/AD cohort and other DS cohorts [15, 46] suggests that TDP-43 is not essential for the development of AD in DS subjects and has a limited contribution to the neuronal degeneration/loss and functional decline in DS/AD.

### Prevalence of α-synucleinopathy and contribution of Lewy bodies to neuronal death in DS/AD subjects

Lewy bodies were reported in 63% of early-onset familial AD cases with known mutations in presenilin or amyloid precursor protein [44]. Estimates of the prevalence of Lewy bodies in DS ranged from one of 12 neuropathologically examined DS cases with Alzheimer-type pathology [39], and in two of 23 people with DS in a study employing antibodies to ubiquitin [68]. In the examined cohort, immunostaining with antibody 4B12 revealed α-synuclein–positive Lewy bodies in neurons in the amygdala in 54.5% of DS/AD subjects 41–59 years of age. The increase in prevalence of Lewy bodies in the amygdala to 75% of DS subjects 61–72 years of age is consistent with the study by Lippa et al. [45] showing increase with age of the prevalence of α-synucleinopathy in DS subjects. However, decrease with age in the number of neurons with Lewy bodies from 861/mm^3^ to 575/mm^3^ (−33%), and decrease of the total number of neurons with Lewy bodies from 337,000 to 201,000 indicates a loss of 39.9% of neurons with Lewy bodies in 75% of DS subjects who developed α-synucleinopathy. Decline in the percentage of neurons with NFTs from 5.3% in the fourth–fifth decades to 3.3% in the sixth decade (−38%) and 39.9% loss of neurons with Lewy bodies suggests some contribution of α-synucleinopathy to neuronal death in DS subjects.

### Unique pattern of β-amyloidosis in DS

In the examined DS cohort, β-amyloidosis was absent in a 26-year-old subject, however, amyloidosis reached final phase 5 in a 28-year-old subject and progressed to Thal phase 5 in all subjects forty or more years of age. A similar pattern of Thal staging of amyloidosis in the DS cohort was reported by Davidson et al. [15], showing phase 1 in 13- and 27-year-old DS subjects, a prevalence of phase 5 in 36- to 47-year-old subjects, and the presence of phase 5 in all DS subjects 51 years of age and older.

The pre-AD stage in subjects 26–41 years of age is characterized by β-amyloidosis with a 2.8% amyloid load. Consistent with expectations, the amyloid load in DS subjects, with a third copy of the APP gene, is approximately 2–3 times greater in DS subjects in the 43–49, 51–59, and 61–72 years of age groups (6.1%, 7.1%, and 6.5%, respectively) than in subjects with LOAD FAST 3–4, 5–6, and 7 (2.1%, 3.8%. and 3.6%, respectively) [94]. Conclusions of this study of brain β-amyloidosis and neurofibrillary degeneration are consistent with Perez et al. [61] observation that in DS despite the early onset and severe brain β-amyloidosis, neurofibrillary degeneration is more closely linked to neuronal loss, functional decline and dementia.

### Diffuse and fibrillar amyloidosis in DS

Brain β-amyloidosis without signs of clinical functional deterioration, reported in some children with DS [43] and in almost all DS subjects 30 years of age and older [100], is characterized by the presence of diffuse nonfibrillar amorphous plaques free of degenerated neuronal processes [101]. The critical change in the pattern of β-amyloidosis and clinical course of AD occurs at the end of the third decade of life of DS subjects, marked by the onset and fast topographic expansion of fibrillar β-amyloid in Congo red– and thioflavin S–positive plaques [100]. This new generation of plaques typical for late-onset AD consists of fibrillar amyloid cores surrounded by several microglial cells, each with a unique amyloid pole with numerous cytoplasmic channels filled with bundles of amyloid fibers. The merger of these bundles of amyloid contributes to the growth of the size of the amyloid core. Leak of fibrillar amyloid at the core periphery results in exposure of surrounding dendrites, axons, and synapses to direct contact with amyloid, leading to degeneration of mitochondria and cytoskeleton and accumulation in swelling segments of neuronal processes and synapses of residues of neuron organelles, cytoskeleton, and paired helical filaments in neurons already affected by neurofibrillary degeneration [96]. Severe injury of neuronal processes and synapses in each fibrillar plaque parallel to an increase in the number of fibrillar plaques and fibrillar amyloid load in adult and aging DS subjects is associated with functional decline and dementia in the fifth and sixth decades of life of DS subjects analogous to the combination of fibrillar amyloidosis, neurofibrillary degeneration, neuronal loss, and dementia in LOAD. The presence of diffuse amyloid deposits without neuronal degeneration in the molecular layer in the cerebellar cortex, striatum, or presubiculum [50, 102] during the entire course of AD confirms mechanistic and functional separation of diffuse and fibrillar amyloid stages of AD pathology in DS subjects [40].

### Cerebral amyloid angiopathy

The study by Head et al. [28] of cerebrovascular pathology including cerebral amyloid angiopathy (CAA), atherosclerosis, and arteriosclerosis revealed CAA in 87.1% of DS subjects, in 72.2% in a sAD cohort, and 18.4% in a control cohort, and an increase of prevalence of severe CAA with age among DS subjects but not in sAD and the control group. In the examined DS cohort, amyloid angiopathy has been detected in all DS subjects 41–72 years of age in all four examined regions, including the EC, CA1, subiculum, and molecular layer in the cerebellar cortex. The study revealed that the number of amyloid-positive vessels reaches a top level in 61- to 72-year-old DS subjects. The increase in the numerical density of amyloid-positive vessels detected in each of four examined brain structures in 61- to 72-year-old DS subjects compared to 41- to 59-year-old DS subjects is consistent with the association between increasing CAA severity and age detected in amyloid precursor protein–overexpressing DS subjects [10, 28].

Growth of deposits of fibrillar β-amyloid in the capillary basal lamina results in perivascular amyloid hemistar formation covered by a mantle of microglial cells, death of endothelial cells, obliteration of affected capillary lumen, microbleeds, and local ischemic changes with neuronal degeneration and death [97, 98]. Amyloidosis of arterioles and arteries is mechanistically separate from capillary amyloidosis. Fibrillar amyloid deposition by smooth muscle cells in the wall of arterioles/arteries results in the degeneration and death of smooth muscle cells in the tunica media, the loss of mechanical strength of artery walls, and the increased risk of disruption of vascular wall and microbleeds [99]. Increase in amyloid angiopathy in all four examined regions in the sixth decade of life of DS subjects indicates that the progression of amyloid angiopathy contributes to regional ischemia, neuronal degeneration and loss, and possibly to functional deterioration. Cerebral amyloid angiopathy leading to vascular dysfunction with impaired vessel constriction and dilation, blood–brain barrier disruption, microhemorrhages, and more severe cerebral amyloid angiopathy in DS/AD than sAD was reported by Head et al. [28]. The association between significant cerebral amyloid angiopathy with deposition of Aβ in the wall of small and medium-size arteries in the cerebral cortex and vascular wall damage with chronic microbleeds [29] or large intracerebral hemorrhages [17] supports the concept of cerebrovascular pathology contributing significantly to AD neuropathology and dementia [34, 81].

### Role of overexpressed minibrain kinase DYRK1A in trisomic DS subjects in synchrony of neurofibrillary degeneration, β-amyloidosis, and functional decline/dementia

Similarities of trajectories of Braak and Thal staging of the onset and progression of both neurofibrillary degeneration and β-amyloidosis and the corresponding onset and progression of dementia in the DS cohort suggest the contribution of common DS-associated underlying molecular mechanisms.

Early onset of β-amyloidosis in DS is considered a result of overexpression of β-amyloid precursor protein (APP), which is encoded by a gene proximal to the DS critical region of chromosome 21. Novel studies revealed a link between overexpression of *Dyrk1A* gene located in the DS critical region of chromosome 21 and neurofibrillary degeneration and β-amyloidosis [21, 37, 47, 92]. Dyrk1A (dual-specificity tyrosine-phosphorylated and regulated kinase 1A) phosphorylates tau protein at Thr212 and several other sites and promotes further phosphorylation by GSK-3β and increases the level of abnormally hyperphosphorylated tau. Overexpression of DYRK1A [18] is associated with hyperphosphorylation of these sites in DS, inhibition of tau’s biological activity, gain of toxic activity leading to sequestration of normal tau and other microtubule-associated proteins, resulting in neuronal degeneration [21] and promotion of tau self-aggregation into NFTs [47, 90, 91]. Moreover, Dyrk1A phosphorylates APP at Thr668, resulting in elevation of the level of phosphorylated APP detected in DS brains [74]. Phosphorylation of APP enhances increasing levels of Aβ40/42 by precursor amyloidogenic cleavage, toxic Aβ oligomer formation, deposition of fibrillar amyloid in plaques, and degeneration of axons, dendrites, and synapses within the fibrillar plaque perimeter. Overproduction of Aβ may up-regulate DYRK1A expression and enhance the contribution of overexpressed DYRK1A to both neurofibrillary degeneration and associated neuronal loss [92] and to the age-associated increase of amyloid load detected in this study.

This study reveals that the range of developmental neuronal deficits contributing to the intellectual deficits of subjects with DS is comparable to the AD-associated neuronal loss contributing to dementia in adult DS subjects. The reduced size of the brain, decrease in neuronal density in the brain of individuals with DS [13, 75, 106], and decreased cell proliferation in the hippocampal dentate gyrus and in the neocortical germinal matrix of fetuses with DS [11, 24] are an effect of decrease in the neurogenesis associated with overexpression of DYRK1A in progenitor cells. DYRK1A is required for the proliferation of distinct neuronal cell types during postembryonic neurogenesis. However, overexpressed DYRK1A may contribute to depletion of the pool of proliferating progenitor cells and may induce premature cell cycle arrest of neurogenic progenitors, leading to a decrease in the number of neurons generated by each progenitor cell [83].

Studies of the contribution of DYRK1A to abnormal brain development and neurogenesis and results of the estimation of developmental neuronal deficits in this study indicate that therapeutic reduction of overexpressed DYRK1A in DS subjects during their development may reduce developmental abnormalities, including defective neurogenesis, which results in a reduced number of neurons and intellectual deficits. The above-cited studies of the contribution of overexpressed DYRK1A to brain β-amyloidosis, neurofibrillary degeneration, neuronal loss, and dementia indicate that therapeutic inhibition of excessive activity of overexpressed DYRK1A in DS individuals may disrupt both pathological pathways and arrest/slow down neurodegeneration, neuronal loss, and related functional decline [92].

## Study limitations and conclusions

The small number of examined DS individuals in the 26–41 and 41–49 years of age groups (4 in each group) limits this part of study to a descriptive approach at this point. Further investigation would require greater tissue acquisition.

The commonly known discrepancy between the reported ubiquity of AD neuropathology in the fourth decade of life of DS subjects and the absence of dementia even at the age of 70 years [27, 77, 109, 110] is partially associated with the challenges of cognitive assessment of DS subjects [5, 51]. The review by Ballard et al. [5] demonstrating that 55% of individuals with DS develop dementia between 40 and 49 years of age and that the percentage of affected people grows to 77% in 60- to 69-year-old subjects discloses the gap between staging of dementia and the ubiquitous brain AD pathology detected postmortem in DS cohorts. The other limitations of clinicopathological correlations are the difficulties of diagnosis and staging of dementia in DS subjects with a broad spectrum of developmental intellectual deficits and the high prevalence of comorbid conditions such as epilepsy [55, 67], hypothyroidism, hypertension, diabetes mellitus, and hyperlipidemia [54], which may contribute to functional decline and onset/aggravation of dementia [77]. These interindividual differences may expand the gap between clinical staging of dementia and postmortem staging of pathology. The difficulties with detection of correlations between clinical staging of dementia in DS and postmortem staging of AD pathology also arise because of the limited accessibility of postmortem material and the common gaps in clinical follow-up for brains donated for research, leading to assessment of dementia based on review of available, not necessarily standardized, medical records, as was applied in this and other studies [28].

Despite the limitations of clinical assessments, this study provides evidence of strong correlations between age and stage of dementia and unbiased measures of the progression of AD pathology in individuals with DS in the fourth, fifth, and sixth decades of life. The study revealed a similar magnitude of developmental deficits of neurons due to trisomy 21 contributing to intellectual deficits and AD-associated neuronal loss leading to dementia. This study demonstrates that age-based staging of pathology in the fourth, fifth, and sixth decades of life in EOAD in DS corresponds to FAST-based staging of pathology in LOAD as well as FAST-based functional decline classified as MCI/mild AD (FAST3-4), moderate and moderately severe AD (FAST 5–6), and severe AD (FAST 7) [94].

## Supplementary Information

**Additional file 1**. Stereological parameters and procedures applied for examination of DS brains.

## Data Availability

The datasets generated and analyzed in this study are available from the corresponding author on a reasonable request. A large portion of the data is presented in 10 Tables and 7 figures/graphs.

## Abbreviations

AD: Alzheimer’s disease
DS: Down syndrome
Aβ: Amyloid-β peptide
APP: A-β precursor protein
EOAD: Early-onset AD
LOAD: Late-onset AD
FAST: Functional Assessment Staging
MCI: Mild cognitive impairment
EC: Entorhinal cortex
ECII: Second layer of the entorhinal cortex
CA: Cornu Ammonis
MBC: Magnocellular basal complex
MRI: Magnetic resonance imaging
NFTs: Neurofibrillary tangles
