# Supplementary material for: Developmental deficits and staging of dynamics of age associated Alzheimer’s disease neurodegeneration and neuronal loss in subjects with Down syndrome
Source: Acta Neuropathol Commun. 2022 Jan 4;10:2. doi: 10.1186/s40478-021-01300-9 (PMC8728914; doi:10.1186/s40478-021-01300-9)
Supplement: Supplementary file 1 — Additional file 1. Stereological parameters and procedures applied for examination of DS brains. [file 40478_2021_1300_MOESM1_ESM.docx]

Supplementary data. Table 1 Stereological Parameters and Procedures Applied for Examination of DS Brains

| Structures/  subdivisions | Mean number of examined  sections  (per case) | Objective  (Volume) | Objective  (Number) | Grid size  (μm) | Test area frame x  height (μm) | Mean number of  counting  spaces  (per case) | Mean  number  of counted neurons  (per case) | CE* |
| --- | --- | --- | --- | --- | --- | --- | --- | --- |
| Entorhinal cortex  (all layers) | 8 | 5x | 40x | 600x600 | 60x60x30 | 518 | 763 | 0.05 |
| Entorhinal cortex  (islands) | 8 | 5x | 40x | 400x400 | 60x60x30 | 83 | 74 | 0.19 |
| CA1  CA2  CA3  CA4 | 11 | 5x | 40x | 800x800  400x400  400x400  600x600 | 60x60x30  60x60x30  60x60x30  100x100x30 | 145  112  99  84 | 243  274  202  234 | 0.08  0.08  0.09  0.09 |
| Subiculum | 12 | 5x | 40x | 600x600 | 60x60x30 | 130 | 235 | 0.07 |
| Amygdala (four nuclei) | 6 | 1.25x | 40x | 1600x1600 | 80x80x30 | 142 | 395 | 0.05 |
| Thalamus | 6 | 1.25x | 40x | 600x600 | 60x60x30 | 386 | 389 | 0.05 |
| Magnocellular  basal complex (Ch1-Ch4) | 11 | 2.5x | 40x | 500x500 | 80x80x30 | 468 | 566 | 0.04 |
| Caudate nucleus  (small neurons) | 14 | 2.5x | 40x | 1400x1400 | 60x60x30 | 281 | 647 | 0.04 |
| Substantia nigra  (pigmented neurons;  pars comp. and ret.) | 6 | 2.5x | 40x | 500x500 | 80x80x30 | 333 | 219 | 0.05 |
| Cerebellum – volume-  mol + gran cell layer and  Purkinje cell number | 8 | 2.5x | 20x | 1800x1800 | 180x180x30 | 1226 | 862 | 0.05 |
| Dentate nucleus | 11 | 1.25x | 40x | 1000x1000 | 180x180x30 | 332 | 780 | 0.05 |

Number of sections examined = mean number of equidistant sections examined per structure/case.

Objective (Volume) = Objective lens (1.25x, 2.5x, or 5x) used for planimetry and volumetry.

Objective (Number) = Objective lens (40x or 20x) used for fractionator to estimate neuronal density and total number of neurons.

*CE = the average predicted Schaeffer coefficient of error in the neuronal counts.
